# Rapid Enrichment of a Native Multipass Transmembrane Protein via Cell Membrane Electrophoresis through Buffer pH and Ionic Strength Adjustment

**DOI:** 10.1021/jacs.3c13579

**Published:** 2024-04-17

**Authors:** Tzu-Tzu Liu, Sin-Han Huang, Ling Chao

**Affiliations:** Department of Chemical Engineering, National Taiwan University, No. 1, Sec. 4, Roosevelt Rd., Taipei 10617, Taiwan

## Abstract

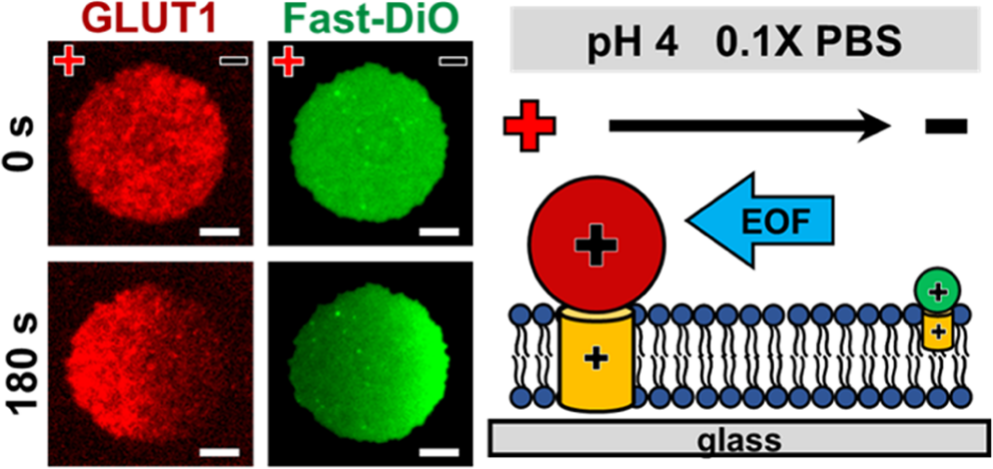

Supported membrane electrophoresis is a promising technique for collecting membrane proteins in native bilayer environments. However, the slow mobility of typical transmembrane proteins has impeded the technique’s advancement. Here, we successfully applied cell membrane electrophoresis to rapidly enrich a 12-transmembrane helix protein, glucose transporter 1 with antibodies (GLUT1 complex), by tuning the buffer pH and ionic strength. The identified conditions allowed the separation of the GLUT1 complex and a lipid probe, Fast-DiO, within a native-like environment in a few minutes. A force model was developed to account for distinct electric and drag forces acting on the transmembrane and aqueous-exposed portion of a transmembrane protein as well as the electroosmotic force. This model not only elucidates the impact of size and charge properties of transmembrane proteins but also highlights the influence of pH and ionic strength on the driving forces and, consequently, electrophoretic mobility. Model predictions align well with experimentally measured electrophoretic mobilities of the GLUT1 complex and Fast-DiO at various pH and ionic strengths as well as with several lipid probes, lipid-anchored proteins, and reconstituted membrane proteins from previous studies. Force analyses revealed the substantial membrane drag of the GLUT1 complex, significantly slowing down electrophoretic mobility. Besides, the counterbalance of similar magnitudes of electroosmotic and electric forces results in a small net driving force and, consequently, reduced mobility under typical neutral pH conditions. Our results further highlight how the size and charge properties of transmembrane proteins influence the suitable range of operating conditions for effective movement, providing potential applications for concentrating and isolating membrane proteins within this platform.

## Introduction

Membrane proteins play pivotal roles in a variety of cellular processes.^1−3^ Beyond their fundamental importance in biological functions, these proteins also serve as diagnostic biomarkers and potential targets for therapeutic interventions against various diseases.^4^ A comprehensive understanding of membrane proteins is imperative for advancing drug development and therapeutic strategies. However, conventional approaches to studying membrane proteins often rely on the use of detergents to solubilize and purify proteins from their lipid environments, which can result in the loss of membrane protein structure and function.^5−9^ Thus, the development of detergent-free methods for purifying and collecting membrane proteins is important for membrane protein research.

Prior studies have introduced supported lipid bilayer electrophoresis as a method to concentrate lipid probes,^10−21^ lipid-anchored proteins,^11,13,14,16,18,22,23^ reconstituted proteorhodopsin,^24^ monohelix transmembrane proteins,^25,26^ and tethered vesicles^27^ within supported lipid membranes. Through the application of an external electric field, charged molecules can be manipulated in terms of both direction and velocity. Furthermore, this platform is well-suited for integration with surface analytical tools, enabling a wide range of applications in bioseparation^14,28^ and biophysical property measurement.^12,21^

However, the separation and concentration of transmembrane proteins in supported membranes are still challenging. One challenge involves the incorporation of native transmembrane proteins into a supported membrane. Some studies have utilized detergents to solubilize membrane proteins and reconstitute them into membranes,^24−26^ but the influence of detergents on protein states after membrane insertion remains unclear. Another obstacle is the slow mobility of native multipass transmembrane proteins,^29−32^ primarily due to the significant drag resulting from the viscous membrane.^12,17,18,27,33−35^ Given that the application of an electric field can lead to joule heating and pH variations through electrolysis,^36,37^ it becomes crucial to collect membrane proteins within a reasonable time frame. The slow mobility can impede the timely collection of transmembrane proteins.

Considering that the buffer pH and ionic strength could significantly impact the electric force and electroosmotic flow,^14,16,38−40^ a pertinent question arises: Can electrophoretic mobility be substantially enhanced by adjusting the buffer conditions? A transmembrane species is partly exposed to the outer aqueous environment and partly embedded in the lipid membrane. Charges in the membrane and in the aqueous environment respond differently to the electric field. Additionally, the electric-field-induced electroosmotic flow can exert a hydrodynamic force on the hydrophilic portion. While several studies have developed models to describe the various forces acting on membrane-bound species during membrane electrophoresis,^14,18,27,41,42^ their models have primarily focused on the migration of labeled lipids or lipid-anchored proteins without considering the electric force on the charged transmembrane region. In addition, no force model has accounted for the effects of both ionic strength and pH on electrophoretic mobilities or drift velocities, factors that have been suggested to play significant roles in influencing electric and hydrodynamic forces.^16,27,39,43^

In this study, we established a supported cell membrane platform with native transmembrane membrane proteins by depositing giant plasma membrane vesicles (GPMVs) on a glass support. GPMVs, derived from cells, offer advantages in preserving the lipid environment and stabilizing transmembrane proteins.^44−47^ We developed a force model that takes into account the electric force and drag force applied not only to the hydrophilic but also to the hydrophobic parts of a transmembrane protein as well as the electroosmotic force. Furthermore, we considered the correlation between ionic strength and pH with the electric force and the electroosmotic force within the model. By employing this model, we demonstrated that under suitable operating conditions, it is possible to enhance the electrophoretic mobility of a native multipass transmembrane protein, glucose transporter 1 (GLUT1), by a remarkable 10^2^ fold. This substantial increase in electrophoretic mobility opens up the possibility of effectively separating membrane proteins from each other within these membrane platforms.

## Experimental Section

### Materials

Dithiothreitol (DTT), paraformaldehyde (PFA), and bovine serum albumin (BSA) were purchased from Sigma-Aldrich (MO). Alexa Fluor 594 goat antirabbit IgG (H + L) and Fast-DiO (ClO4 (3,3′-Dilinoleyloxacarbocyanine Perchlorate)) were purchased from Invitrogen (MA). Antiglucose transporter GLUT1 antibodies (ab15309) were purchased from Abcam (Cambridge). Glass coverslips were acquired from VMR (PA). Poly(dimethylsiloxane) (PDMS: Sylgard 184) was purchased from Corning (NY).

### Preparation of Giant Plasma Membrane Vesicles (GPMVs)

HeLa cells were cultured in Dulbecco’s Modified Eagle Medium (DMEM), supplemented with 10% fetal bovine serum (FBS) and 1% antibiotic antimycotic solution. Cells were maintained at 37 °C in 5% CO_2_ and incubated for 2 days. The cultured HeLa cells were rinsed with 10 mM phosphate-buffered saline (PBS) buffer (137 mM NaCl, 2.7 mM KCl, 10 mM Na_2_HPO_4_, and 2 mM NaH_2_PO_4_, pH 7.4). For the experiment requiring a lipid probe, a Fast-DiO solution (5 μg/mL in PBS buffer) was added and incubated at 4 °C for 10 min. Then the cells were rinsed with GPMV buffer (2 mM CaCl_2_, 150 mM NaCl, and 10 mM HEPES, pH 7.4). Blebbing buffer (25 mM PFA, 2 mM DTT in GPMV buffer) was added to the culture dish. After the blebbing buffer was added to the culture dish, cells were maintained in a 37 °C, 5% CO_2_ incubator for 1 h. After that, GPMVs were collected to prepare supported cell membranes.

### Preparation of Supported Cell Membranes in Microchannels

A microfluidic channel was created by using PDMS. The length of the linear channel was 1.3 cm, with a 1 mm width and 100 μm height. To form the supported cell membrane, glass coverslips were cleaned with argon plasma for 12 min. After plasma treatment, a PDMS well was attached to the glass substrate. The GPMV solution was added to the PDMS well. After 30 min, the sample was rinsed with GPMV buffer to wash off the unruptured GPMVs.

### Procedure of Immunofluorescence Staining

The cell membrane was incubated in blocking buffer (5% w/v BSA in PBS buffer) at room temperature for 1 h. After the blocking procedure, the sample was rinsed with PBS to remove excessive BSA. 1.5 μg/mL amount of antiglucose transporter GLUT1 antibody (diluted in 0.5% BSA in PBS) was added to the sample and incubated at room temperature for 1 h. After incubation, the sample was rinsed with PBS buffer to remove unbound antibodies. Then, 8 μg/mL antirabbit secondary antibody conjugated with Alexa 594 (diluted in 0.5% BSA in PBS) was added to the sample and incubated at room temperature for 1 h.

### Supported Membrane Electrophoresis

Before electrophoresis, the sample was rinsed with the electrophoresis buffer, and the PDMS-based microchannel was placed on the supported cell membrane for the device assembly. The electrophoresis buffer was diluted from a typical PBS buffer, and NaOH or HCl was used to adjust the pH value. The amount of NaOH and HCl added was considered in the calculation of Debye length. Electrophoresis was performed with an electric field of 38 V/cm. An inverted microscope (Olympus IX83, Japan) was used to capture real-time images. The images were taken by using Cell Sense software. The images of the GLUT1 complex were taken every 10 s for 1 min and then every 20 s for 4 min, and the images of Fast-DiO were taken every 2 s for 30 s and then every 5 s for 1.5 min. ImageJ (NIH, MD) and MATLAB (MathWorks, MA) software were used to process the images.

## Results and Discussion

### Movement of GLUT1 Complex and Fast-DiO in Membrane Electrophoresis Platform

**Figure 1:**
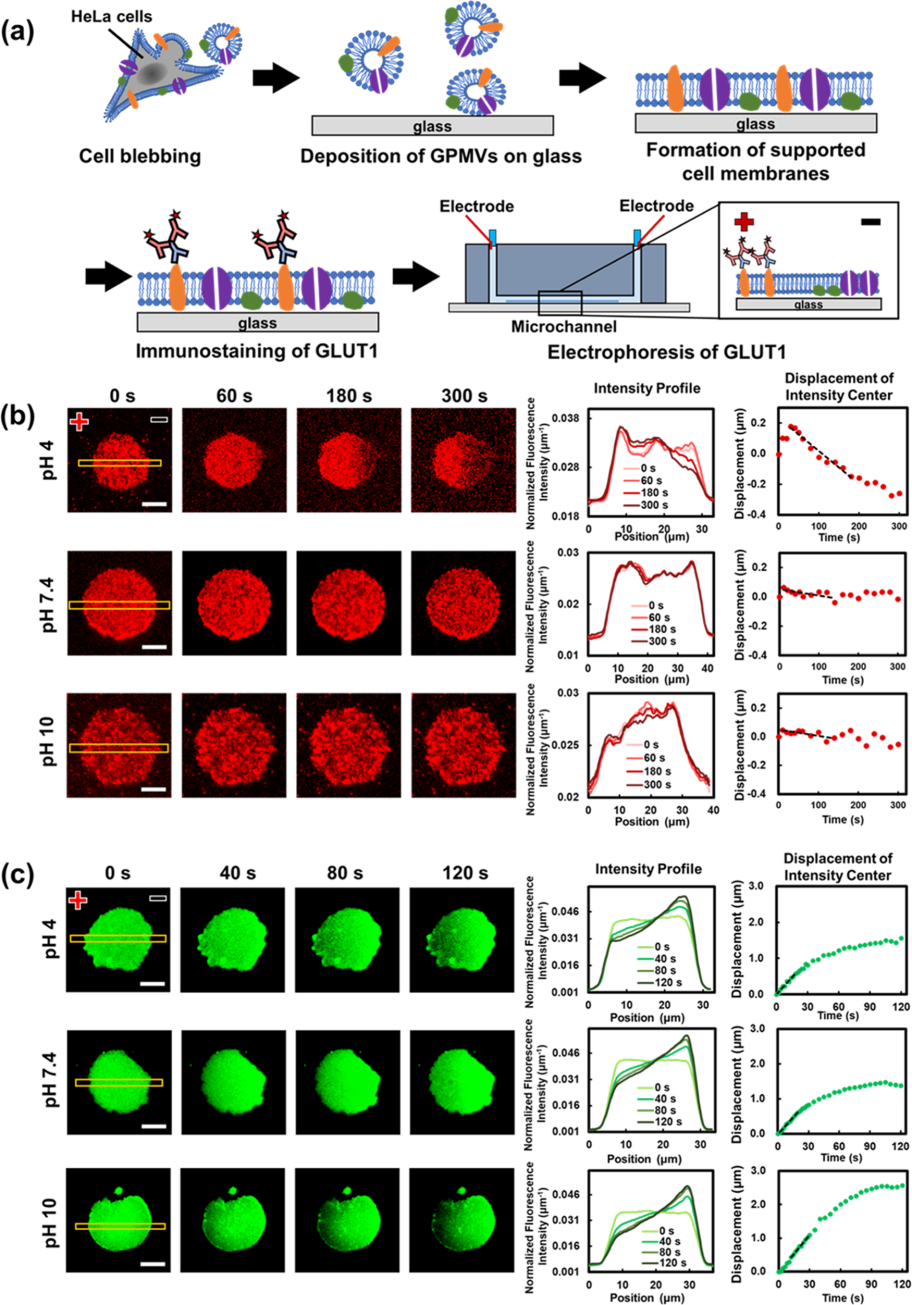
(a) Schematic representation of the preparation of a supported cell membrane electrophoresis platform. (b) (Left) Fluorescence images of the GLUT1 complex (0.1× PBS) within membrane patches at various pH values under membrane electrophoresis, with a 10 μm scale bar. (Right) The corresponding normalized intensity profiles and displacement of fluorescence intensity center at different pH values. (c) (Left) Fluorescence images of Fast-DiO (0.1× PBS) at different pH values under membrane electrophoresis, with a 10 μm scale bar. (Right) The corresponding normalized intensity profiles and displacement of fluorescence intensity center at different pH values.

We established a supported membrane platform to investigate the response of membrane proteins when they were subjected to an applied electric field. To create this platform, we collected giant plasma membrane vesicles (GPMVs) through cell blebbing and allowed them to deposit onto a glass surface, forming supported cell membranes (depicted in Figure 1a). Once the supported cell membrane formed, and the target proteins were labeled, we integrated this supported membrane into a flow device and initiated electrophoresis by applying an electric field.

In this study, we selected GLUT1 as our target protein due to its abundant presence in HeLa cell membranes. Figure 1b (left) presents the electrophoresis results of labeled GLUT1 within GPMV patches in 0.1× PBS at pH 4, 7.4, and 10. Intriguingly, we observed that the labeled GLUT1 moved toward the anode (leftward) at pH 4 during the electrophoresis process, while the migration velocities at pH 7.4 and pH 10 were significantly slower compared to the one at pH 4. To quantitatively assess the migration of the GLUT1 complex, we introduced the concept of drift velocity, which is based on the average rate of motion of effective mobile membrane species. Detailed information and calculation methods for the drift velocity are provided in the Supporting Information (SI). Figure 1b (right) illustrates how fluorescence intensity profiles and the displacement of the fluorescence intensity centers evolved over time. Specifics on obtaining normalized intensity profiles and displacement measurements can also be found in the SI.

The substantial migration of the GLUT1 complex toward the anode at pH 4, despite its positive charge as estimated from the amino acid sequence (Figure S3 and Table S4), was out of our expectations. According to conventional electrostatic principles, a positively charged molecule should move in the direction of the electric field (toward the cathode). This result suggests that the presence of forces other than the electric force plays a crucial role in driving its migration.

To further study the migration of membrane species within the membrane, we also introduced a fluorescent lipid probe, Fast-DiO, into the membrane and monitored its migration during membrane electrophoresis. The images on the left in Figure 1c reveal significant migration of Fast-DiO molecules toward the cathode under all three pH conditions. This aligns with the reported charge of Fast-DiO molecules, which is known to be +1.^48^ The displacement profile on the right demonstrates that Fast-DiO molecules migrate faster than the GLUT1 complex, and the drift velocity of Fast-DiO exceeds that of the GLUT1 complex. This observation is reasonable considering that the transmembrane portion of Fast-DiO is considerably smaller than that of the GLUT1 complex, leading to reduced drag forces exerted by the viscous cell membrane that hinders migration. Furthermore, unlike the GLUT1 complex, the migration rates of Fast-DiO are observed to be similar across all three pH conditions. These contrasting behaviors of these two molecules imply that we could identify suitable operational conditions for separating membrane species with varying sizes and charges through membrane electrophoresis.

### Model to Correlate the Membrane Protein Properties to the Electrophoretic Mobility

**Figure 2:**
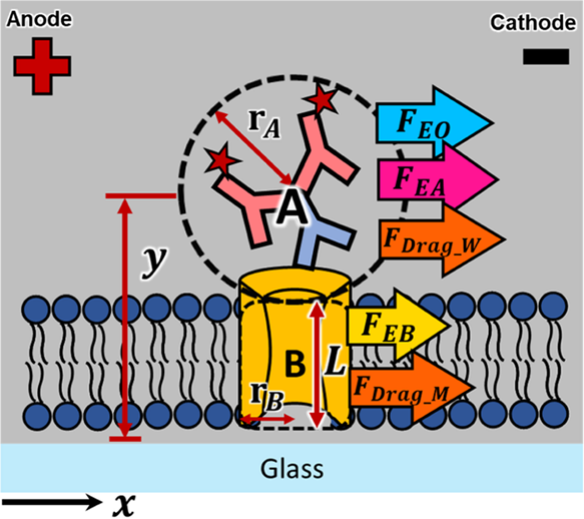
Proposed force model of a transmembrane protein labeled with antibodies in the supported cell membrane.

For the potential application of separating various membrane species, we developed a model aimed at establishing a relationship among the sizes of membrane species, the operational conditions, and the protein migration velocities. Illustrated in Figure 2 is the force model we devised for a labeled transmembrane protein within the supported cell membrane. We conceptualized the membrane protein complex as consisting of two distinct segments: portion A, situated in an aqueous environment, and portion B, residing within the membrane. Portion A represents the hydrophilic region of the transmembrane protein along with the clustering of labeling antibodies, while portion B denotes the hydrophobic transmembrane region of the membrane protein. To simplify our model, we approximated portion A as a spherical entity and portion B as a cylindrical one. In the model, we considered that there are five major forces exerting on the membrane protein during the membrane electrophoresis: the electric force on portion A (*F*_EA_), the electric force on portion B (*F*_EB_), the hydrodynamic force by the induced electroosmotic flow (*F*_EO_), the drag force from the membrane (*F*_drag_M_), and the drag force from the aqueous solution (*F*_drag_W_).

#### Electroosmotic Force on Portion A

Many studies have shown that a hydrodynamic force can drive the migration of membrane species in lipid membranes.^23,49−51^ Although we did not apply forced convection to the system, applying an electric field can induce an electroosmotic flow. An electroosmotic flow is the motion of liquid induced by an applied potential across a capillary tube or microchannel with a surface charge.^39^ When an electric field is applied, the counterions in the electric double layer start to migrate under the influence of the electric field. The momentum of the counterions in the electric double layer is transferred to the surrounding liquid, leading to an electroosmotic flow.^39,52^ If we consider an electroosmotic flow in a cylindrical pore, the velocity is given by^53^
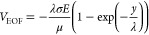
1where *V*_EOF_ is the velocity of the electroosmotic flow in the direction of the applied electric field, σ is the surface charge density, *E* is the electric field, μ is the viscosity of the aqueous medium, *y* is the distance from the charged surface, and λ is the Debye length. Debye length is often used to describe the thickness of the electric double layer,^17,52^ and the expression is given by
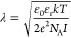
2where λ is the Debye length, ε_0_ is the permittivity of free space, ε_*r*_ is the dielectric constant, *k* is the Boltzmann constant, *e* is the elementary charge, *N*_A_ is the Avogadro constant, and *I* is the ionic strength of the aqueous medium.

The hydrodynamic force acting on the entire portion A can be determined by integrating the shear stress resulting from the electroosmotic flow across the entire surface. To streamline the computation, we simplify portion A as a sphere and apply the electroosmotic force to the center of mass of this spherical approximation. Applying Stoke’s law, the driving force induced by the electroosmotic flow on section A can be expressed as eq 3.(17,27,39)
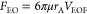
3where *r*_A_ is the hydrodynamic radius of portion A.

#### Electric Force on Portion B

Since GLUT1 and the antibodies are typically charged, the electric field exerts forces on both portion A and portion B of the GLUT1 complex. Because portion B is surrounded by the cell membrane, we assumed that the environment of portion B is free of electrolytes. Without the existence of electrolytes, the force *F*_EB_ is expressed by the product of the electric field and the net charge of portion B.^27,39,54,55^
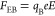
4where *e* is the charge, *E* is the electric field, and *q*_B_ is the net charge of portion B.

#### Electric Force on Portion A

The force exerted by the electric field on portion A is more complicated than that on portion B. Portion A is surrounded by ions in the aqueous medium. When an electric field is applied, the surrounding electrolytes are also affected by the electric field. The electrolyte distribution around portion A can also provide an additional electric field to portion A.^54^ Poyton and Cremer have used Henry’s function to describe the electric force applied to a lipid probe with a charged headgroup.^12^ Henry derived a formula to express the electrophoretic mobility of a spherical particle in solutions,^38^ which is given by
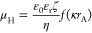
5where μ_H_ is the mobility of the particle, ζ is the ζ-potential of the charged particle, κ is the reciprocal of the Debye length, and *f*(*κr*_A_) is Henry’s function. If *κr*_A_ approaches infinity, the value of Henry’s function is close to 1; if *κr*_A_ approaches 0, Henry’s function is close to 2/3. Ohshima proposed an approximate expression for Henry’s function,^56^ which is given by
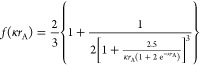
6

To express the net force exerted on portion A, we started by considering a charged sphere free to move in aqueous medium. After an electric field is applied, the particle starts to migrate and finally moves at a steady velocity. The steady velocity indicates that the Stokes drag balances the net force from the electric field, including the force exerted on the charges of the particles by the electric field and the electrophoretic retardation force by the ionic environment.^42^ By replacing drift velocity in the formula of Stokes drag with the expression of mobility in eq 5, we expressed the electric force exerted on portion A (*F*_EA_) by the following equation^27,39^
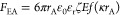
7

#### Drag Forces on Portions A and B

We hypothesized that the hydrodynamic friction on portion A resulting from the relative motion between the electroosmotic flow and the GLUT1 complex follows the Stokes’ law,^57^ and can be expressed as eq 8
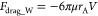
8where *F*_drag_W_ is the drag force exerted on portion A, *r*_A_ is the hydrodynamic radius of portion A of the GLUT1 complex, μ is the viscosity of water, and *V* is the drift velocity of the GLUT1 complex.

During the migration of the protein complex, the supported cell membrane exerted a drag force on portion B of the GLUT1 complex. We also assumed that portion B is surrounded by the cell membrane medium and expressed the drag force by Stoke’s law.^57^ Previous studies have considered the membrane-embedded portion as a cylinder to calculate the drag from the membrane. The formula for a cylinder is given by eq 9.(17,27)
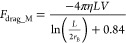
9where *F*_drag_M_ is the drag force exerted by the cell membrane, η is the viscosity of the membrane, *r*_B_ is the radius of the cylinder, and *L* is the height of the cylinder. Combining both of the drag forces exerted on the GLUT1 complex, we get eq 10
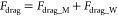
10

#### Force Balance to Correlate Drift Velocities and Electrical Properties

When the velocity of a membrane protein reaches steady state, the net force on the complex is zero. The force balance formula can be expressed in eq 11.
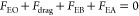
11

After expressing ζ in the form of Debye length calculated from the Poisson equation and Debye–Hückel approximation,^43^ we inserted eqs 3, 4, 7, and 10 into eq 11 to obtain eq 12
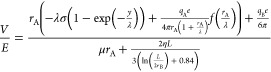
12

Equation 12 clearly shows that electrophoretic mobility is a function of the Debye length and the three parameters specifying the electrical properties in our system, including the surface charge density σ, the net charge of portion A, *q*_A_, and the net charge of portion B, *q*_B_. We reorganized eq 12 in the form of
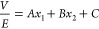
13where A, B, and C are parameters related to the electrical properties and *x*_1_, *x*_2_ are those associated with the Debye length. The complete expressions of these parameters are described in eqs 14–18.


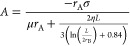
14
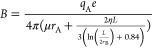
15
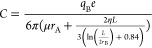
16
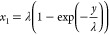
17
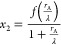
18

According to the equation, we prepared electrophoresis buffers with different ionic strengths to alter the Debye length and experimentally measured the electrophoretic mobility. The fitting of the electrophoretic mobility to the Debye length can result in the fitted parameters A, B, and C, which have information about the electrical properties, including the surface charge density, σ, the net charge of portion A, *q*_A_, and the net charge of portion B, *q*_B_.

### Electrophoretic Mobility of GLUT1 Complex Varying with pH and Ionic Strength

Our model demonstrates that electrophoretic mobility is influenced by several key factors, including the Debye length, geometric attributes of the membrane species, and electrical characteristics of the system. For a specific protein with certain geometric properties, the Debye length, and electrical properties of the system, the magnitudes and directions of forces have a significant impact, thereby exerting a substantial influence on the electrophoretic mobility. The Debye length is intricately linked to the ionic strength of the buffer solution used and the pH-dependent electrical properties of the protein and the membrane surface.

**Figure 3:**
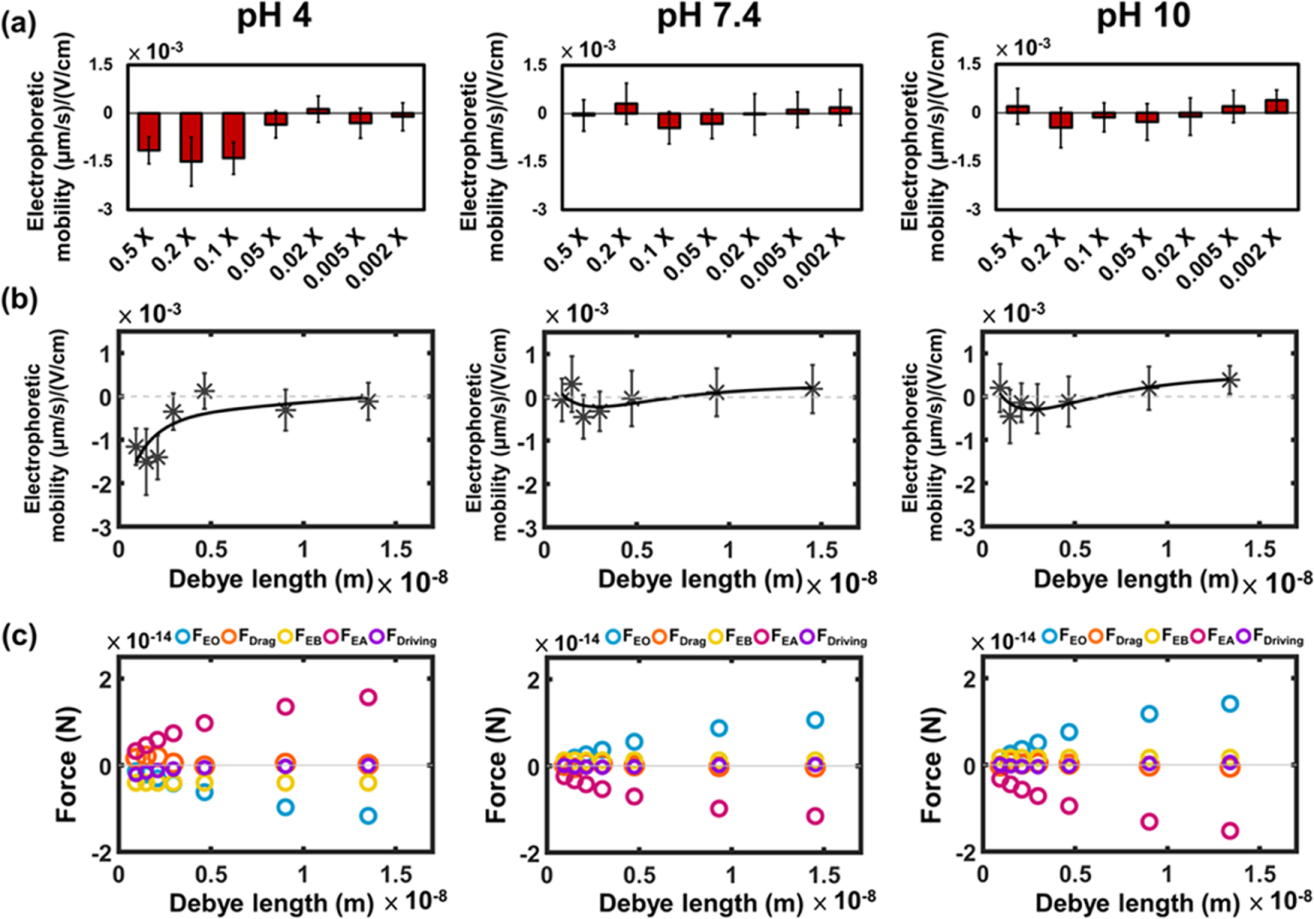
(a) Electrophoretic mobilities of the GLUT1 complex at pH 4, pH 7.4, and pH 10 under various PBS buffer concentrations (*n* = 30 from three independent experiments; 10 patches per experiment). (b) The results of nonlinear curve fitting at pH 4, 7.4, and pH 10. (c) Calculated forces that acted on the GLUT1 complex at pH 4, 7.4, and pH 10. Electroosmotic force (*F*_EO_): blue circle. Total drag force (*F*_drag_M_ + *F*_drag_W_): orange circle. Electric force on portion B (*F*_EB_): yellow circle. Electric force on portion A (*F*_EA_): pink circle. Total driving force (*F*_EO_ + *F*_EA_ + *F*_EB_): purple circle.

To examine whether the electrophoretic mobility can be well described by our model, we conducted experimental measurements of the drift velocity of the GLUT1 complex under an electric field of 38 V/cm at varying ionic strengths and pH levels. In Figure 3a, the electrophoretic mobility exhibits changes with variations in ionic strength at different pH levels. At pH 4, the electrophoretic mobility can reach −0.0014 ± 0.0005 (μm/s)/(V/cm) in a 0.1× PBS solution, but this value decreases significantly as the ionic strength decreases. At pH 7.4 and 10, the electrophoretic mobilities remain in the order of 10^–4^ (μm/s)/(V/cm) over the range from 0.5× to 0.002× PBS.

Figure 3b illustrates the results of fitting our experimentally measured data to eq 13. The parameters obtained through this fitting process enable us to calculate the electrical properties of our system, including the surface charge density (σ), the effective charge of portion A (*q*_A_), and the effective charge of portion B (*q*_B_), as indicated in eqs 14–16. The necessary constants for these calculations are summarized in Table S3, and the derived electrical properties are presented in Table 1.

**Table 1:** Fitted Results and Predicted Values of the GLUT1 Complex Electrical Properties

	pH 4	pH 7.4	pH 10
effective σ (C/m^2^)	0.0027	–0.0024	–0.0032
effective *q*_A_ (−)	38.77	–27.95	–37.70
effective *q*_B_ (−)	–6.56	2.10	2.68
estimated *q*_A_ (−)	55.64	–0.71	–20.20
estimated *q*_B_ (−)	15.42	–3.24	–25.20

Furthermore, we calculated the magnitude and direction of the forces acting on the GLUT1 complex based on the fitting results, as depicted in Figure 3c. Due to the positive surface charge at pH 4, the electroosmotic flow is directed toward the anode, resulting in a hydrodynamic force applied in the negative direction. *q*_A_ is found to be positive, and the electric force exerted on the A portion is in the positive direction, while *q*_B_ is slightly negative, and the electric force on the B portion is in the negative direction. The membrane drag force (*F*_drag_M_) is exerted on the complex in the opposite direction of the electrophoretic mobility.

Upon application of an electric field, the effective electric forces (*F*_EA_, *F*_EB_) and the electroosmotic force (*F*_EO_) start to appear and drive the movement of the membrane protein. As mobility increases, drag forces also rise in proportion to velocity until they ultimately balance the driving force. When the net force reaches zero, the mobility no longer increases, and the system reaches a steady state. Consequently, the magnitude of the net driving force determines the mobility of a membrane protein at the steady state. Notably, in regions of high ionic strength at pH 4, the magnitude of the net driving force is considerably larger than under other conditions, resulting in a higher electrophoretic mobility, wherein the membrane protein moves toward the anode due to the negative net driving force.

### Electrophoretic Mobility of Fast-DiO Varying with pH and Ionic Strength

To further validate our model, we discussed the electrophoretic results of Fast-DiO, a lipid probe with a well-known charge and structure, with our model. Fast-DiO possesses a single positive charge and a structure resembling a cylinder embedded in the membrane with a radius of approximately 0.4 nm and a height of about 2 nm.

**Figure 4:**
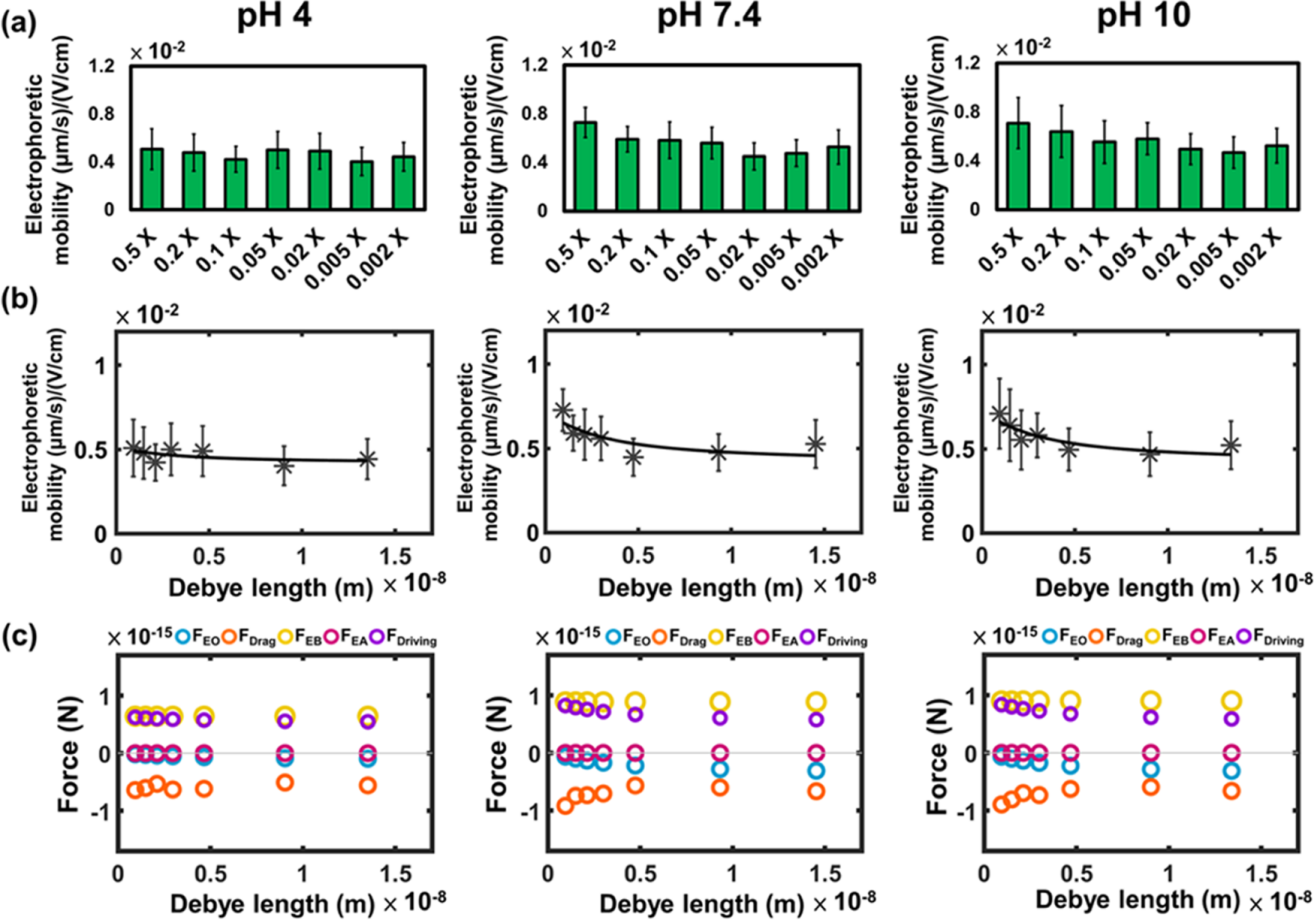
(a) Electrophoretic mobilities of Fast-DiO at pH 4, pH 7.4, and pH 10 under different PBS buffer concentrations (*n* = 60 from six independent experiments; 10 patches per experiment). (b) The result of nonlinear curve fitting at pH 4, 7.4, and pH 10. (c) Calculated forces that acted on the Fast-DiO at pH 4, 7.4, and pH 10. Electroosmotic force (*F*_EO_): blue circle. Total drag force (*F*_drag_M_ + *F*_drag_W_): orange circle. Electric force on portion B (*F*_EB_): yellow circle. Electric force on portion A (*F*_*EA*_): pink circle. Total driving force (*F*_EO_ + *F*_EA_ + *F*_EB_): purple circle.

Figure 4a presents the experimentally determined electrophoretic mobilities of Fast-DiO at varying ionic strength and pH. As anticipated, the positive charge propelled it toward the cathode. The smaller portion B of Fast-DiO within the membrane, compared to the GLUT1 complex, resulted in reduced drag resistance and subsequently faster electrophoretic mobilities. An intriguing observation is that as the ionic strength increased or the Debye length decreased, the electrophoretic mobilities exhibited a slight increment, a phenomenon we discuss below.

Figure 4b shows the results obtained from fitting our experimental data to eq 13. The value of *r*_B_ of Fast-DiO was set as 0.4 nm based on the structural characteristics. Although we expect only a minor extension (portion A) of a lipid probe beyond the membrane, we identified both *r*_A_ and the resulting electroosmotic forces as critical influences. This is because we noted an increase in electrophoretic mobility corresponding to rising ionic strength. In our model, the electric force acting on portion B remains unaffected by changes in the ionic strength, while the electric force on portion A diminishes with increasing ionic strength. Thus, the only conceivable explanation for the observed increase in the electrophoretic mobility or net driving force with ionic strength is the pivotal role played by the electroosmotic force. Despite the reduction in magnitude of the electroosmotic force with increasing ionic strength, its direction opposes the electric force, resulting in an increase in the net driving force. The significant electroosmotic force implies the existence of a substantial portion of A, where electroosmotic flow is applicable. Since the size of portion A remains uncertain, we varied the value of *r*_A_ from 0 to 1 nm and determined that a value of *r*_A_ ∼ 0.5 nm enables a nice fit to the observed electrophoretic mobilities under the three pH conditions. We applied the same fitting process as shown in the previous subsection to obtain the electrical properties of Fast-DiO. Since most of the lipid probe is embedded in the membrane, we assumed that *q*_A_ = 0 and obtained *q*_B_ values through the fitting procedure. The obtained effective surface charge density (σ) values are 0.0006, 0.0019, and 0.0019 C/m^2^ at pH 4, pH 7.4, and pH 10, respectively. The corresponding *q*_B_ values (1.04, 1.44, and 1.46) align closely with the Fast-DiO characteristic single positive charge.

In Figure 4c, we present the calculated forces based on the fitting results of the Fast-DiO electrophoretic mobilities. As anticipated, the electric force on portion B remains positive and is unaffected by changes in ionic strength. The electric force on portion A is positive and exhibits a decrease with increasing ionic strength. Simultaneously, the electroosmotic force is negative, and its magnitude decreases with increasing ionic strength. This combination results in an increase in the net driving force with ionic strength in the direction of the electric field, aligning with the experimental observation of increasing electrophoretic mobilities with higher ionic strength.

### Using Membrane Electrophoresis to Separate the GLUT1 Complex and Fast-DiO in Cell Membrane Patches

We further demonstrated our ability to separate distinct membrane species based on their different migration responses. Our selection of specific conditions was informed by the results presented in Figures 3 and 4. Specifically, we opted for a pH of 4 and a 0.1× PBS concentration, conditions under which the GLUT1 complex and Fast-DiO exhibit a substantial disparity in electrophoretic mobilities. In contrast, we also selected a typical condition characterized by a pH of 7.4 and a 0.005× PBS concentration, where the difference in electrophoretic mobilities between GLUT1 complex and Fast-DiO is small.

**Figure 5:**
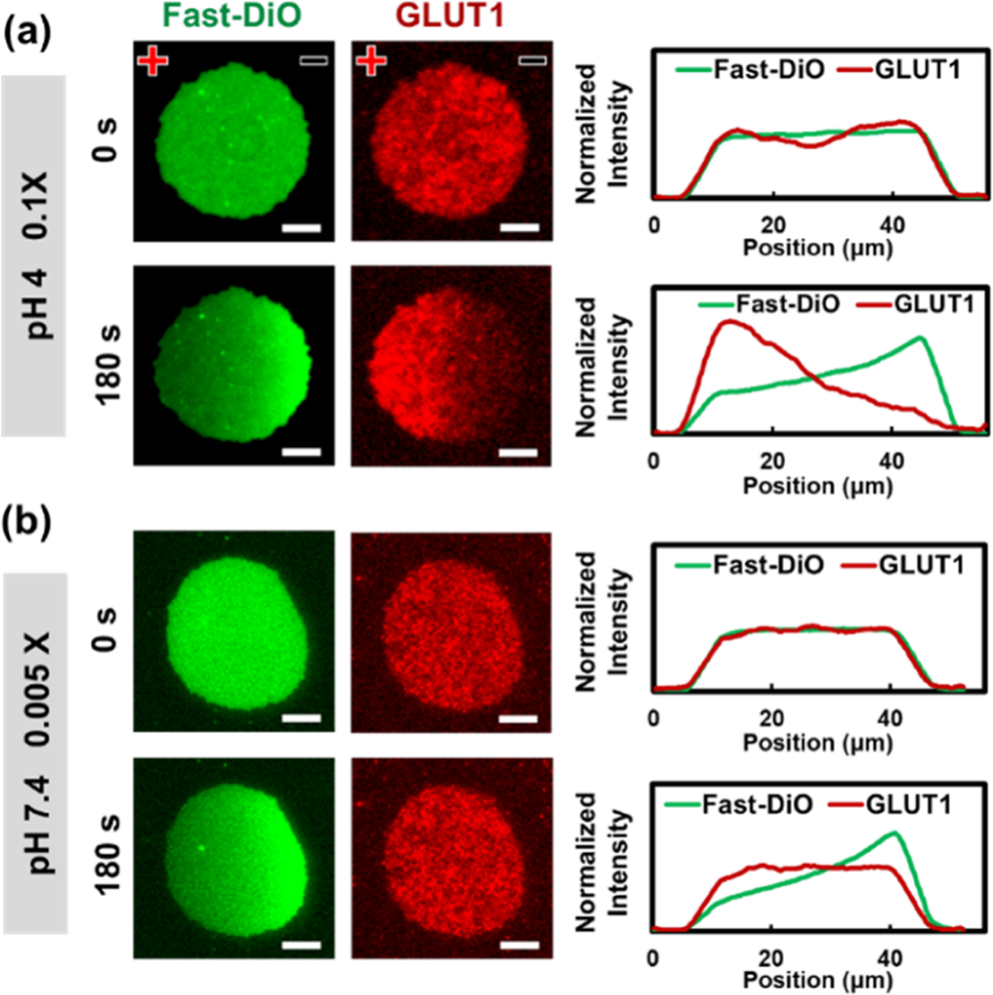
(Left) Fluorescence images depicting the GLUT1 complex and Fast-DiO within cell membrane patches, showcasing their behaviors in two distinct buffer environments during membrane electrophoresis. Scale bar: 10 μm. (Right) Normalized intensity profiles of the GLUT1 complex and Fast-DiO captured at 0 and 180 s after the initiation of membrane electrophoresis.

Figure 5 (left) shows the separation results. At pH 4 with a 0.1× PBS concentration, Fast-DiO migrated significantly toward the anode, while the GLUT1 complex exhibited notable cathodic migration, aligning with our expectations. Consequently, a mixture of Fast-DiO and the GLUT1 complex was effectively separated into two distinct groups positioned on either side of a membrane patch. Conversely, at pH 7.4 with a 0.005× PBS concentration, Fast-DiO migrated toward the cathode, while the GLUT1 complex displayed negligible migration. These results underscore the critical importance of selecting appropriate buffer conditions for achieving the effective separation of membrane species in supported membrane electrophoresis.

### Examining the Rationality of the Obtained Effective Electrical Properties

It is still challenging to obtain the charges of the transmembrane proteins. Conventional methods, such as membrane-confined electrophoresis (MCE) or electrophoretic light scattering (ELS), rely on using detergents to solubilize and purify proteins from their lipid environment due to the amphiphilic nature of transmembrane proteins.^5^ However, detergent adsorption on the protein could alter the measured surface charge. In addition, it is also difficult to know the individual charge information on the hydrophilic portion and hydrophobic portion.

In this study, we acquired the effective electrical properties of the GLUT1 complex at different pH values by fitting the electrophoretic mobility to our developed model. The effective charge of portion A (*q*_A_) from the curve fitting was 38.77, – 27.95, and −37.70 at pH 4, pH 7.4, and pH 10, respectively. The value of *q*_B_ was −6.56, 2.10, and 2.68 at pH 4, 7.4, and 10, respectively. To discuss whether these values predicted from the model fitting are reasonable, we estimated GLUT1′s charge based on its amino acid sequence. Protein charges primarily result from ionizable side chains of specific amino acids,^58,59^ influenced by the surrounding environment pH. The amino acid sequences of GLUT1 from the Uniprot database^60^ and how we calculated the charge of the cytoplasmic, transmembrane, and extracellular regions of GLUT1 at different pH levels based on the p*K*_a_ values of the ionizable amino acids are shown in the SI.

In our model, portion A represents the hydrophilic part of the protein complex, while portion B represents the hydrophobic part. Considering that most membrane patches have their cytosolic side facing the bulk solution,^46^ the effective charge of portion A should account for the cytosolic part of GLUT1, including the labeled antibodies (rabbit IgG and goat IgG). The net charge of different monoclonal IgGs at varying pH values is derived from the literature,^61^ with measured charges of 7.05 at pH 5 and −7.11 at pH 7.4. For portion B, we consider both the transmembrane and extracellular parts, assuming that the extracellular part is less influenced by mobile electrolytes based on the properties of the thin water layer under the supported lipid bilayer.

Table 1 presents a comparison between the effective charges of portions A and B obtained from the model fitting and the charges estimated from the amino acid sequence. The effective *q*_A_ values at the three pH levels align closely with those estimated from the amino acid sequence. However, the effective *q*_B_ values diverge from the estimated values. Notably, at low pH, a negative charge is observed, which transitions to a positive charge at high pH, contradicting the expected acid–base dissociation pattern.

Our hypothesis attributes this phenomenon to the induced electric field by the polarization of rapidly moving membrane species. We have noted that the displacement–time curves for the GLUT1 complex have a typical pattern: an initial, brief startup phase is followed by a pronounced linear change in the midphase and then a phase characterized by more gradual change. This pattern indicates a difference in migration speeds among the various membrane species. In the cell membrane, components with a smaller hydrophobic portion B experience less drag and thus have higher mobility and faster migration rates compared with those with a larger portion B. When an electric field is applied, the smaller charged membrane species can quickly migrate and accumulate on one side of a membrane patch. This rapid accumulation of smaller components may create an additional electric field, which in turn affects the movement of larger, slower-moving membrane proteins.

**Figure 6:**
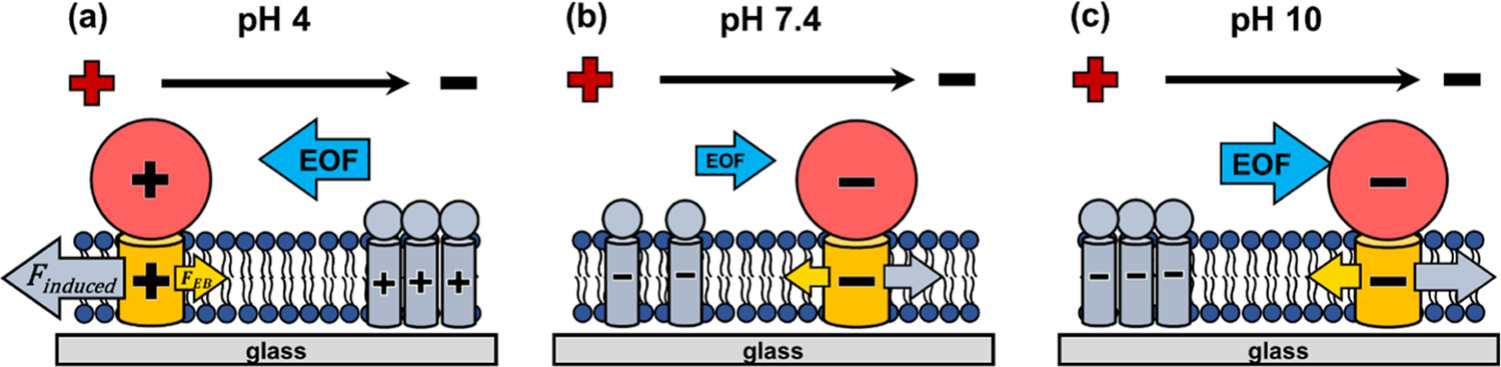
Schematic illustration of how rapidly moving charged membrane species affect the migration of the GLUT1 complex at different pH levels: (a) pH 4, (b) pH 7.4, and (c) pH 10. The yellow arrow is the intrinsic electric force exerted on portion B due to the intrinsic charge of portion B. The gray arrow is the induced electric force caused by the polarization of the rapidly moving membrane species. The effective electric force applied on portion B is the summation of the intrinsic electric force and the induced electric force. The blue arrow indicates the electroosmotic flow.

Figure 6 further depicts the different scenarios at the three pH levels. At pH 4, with the predominance of positively charged membrane species, a polarization of small positive components toward the cathode side of the membrane patch might quickly occur before the slowly moving membrane proteins have significant movement. The polarization might induce an additional electric force to the positively charged GLUT1 complex toward the anode as shown in Figure 6a. The effective *q*_B_ value is determined by the total electric force acting on portion B. Should this induced electric force surpass the force generated by portion B charge, the resultant net electric force on portion B would be directed toward the anode. This accounts for the observed negative effective q_B_ value. In contrast, at pH 10, when the membrane species are primarily negatively charged, a polarization of the small negative components toward the anode side may occur, as depicted in Figure 6c. This leads to an additional electric force toward the cathode on the negatively charged GLUT1 complex. If this induced force exceeds the inherent electric force, the net electric force on portion B is toward the cathode, resulting in a positive effective *q*_B_ value. At pH 7.4, the charges on membrane species tend to be less negative than at pH 10, reflective of the p*K*_a_ values of common amino acids and lipids found in cell membranes. Therefore, both the induced electric force toward the cathode and the intrinsic electric force of portion B might be smaller. It is likely that the net electric force on portion B is still toward the cathode for the GLUT1 complex, leading to the obtained positive effective *q*_B_. Notably, the potential impact on effective *q*_A_ values is also possible, although it is expected to be less pronounced due to the screening effects of electrolytes in the solution.

Despite the ongoing mutual interaction between various membrane species, our data reveal a section of linear movement in the displacement–time curve for the GLUT1 complex. This apparent linear movement during the midphase indicates a pseudosteady state, wherein the electric forces resulting from the redistribution of membrane species become constant, and the total force on the GLUT1 complex nears zero. Since the effective charges (*q*_A_, *q*_B_) of the GLUT1 complex are obtained by fitting the electrophoretic mobility at this midphase pseudosteady state, they contain the effect from the induced electric forces by the transient redistribution. On the other hand, the displacement–time curve demonstrates that Fast-DiO moves swiftly, usually achieving its steady state soon after the electric field is applied. Hence, the steady-state electrophoretic mobility of Fast-DiO is often measured at an early stage with minimal polarization effects, which might be why the effective *q*_B_ values of Fast-DiO more accurately reflect its intrinsic single positive charge.

Table 1 also shows the effective surface charge density (σ) obtained through curve fitting, showing values of 0.0027, −0.0024, and −0.0032 C/m^2^ at pH 4, pH 7.4, and pH 10, respectively. The effective surface charge at pH 7.4 is at the same scale as the glass surface charge reported in previous studies.^40^ The values at pH 4 and pH 10 also align with acid–base dissociation principles,^62^ supporting the feasibility of using the model to obtain the effective surface charge density.

### Prediction of the Electrophoretic Mobility of Membrane Species

Our model elucidates that a transmembrane species’ electrophoretic mobility is intricately governed by the interplay of various forces acting upon it. These forces are subject to substantial modulation by both intrinsic protein properties, such as charges and the sizes of hydrophilic portions, and external buffer conditions, including pH and ionic strength.

**Figure 7:**
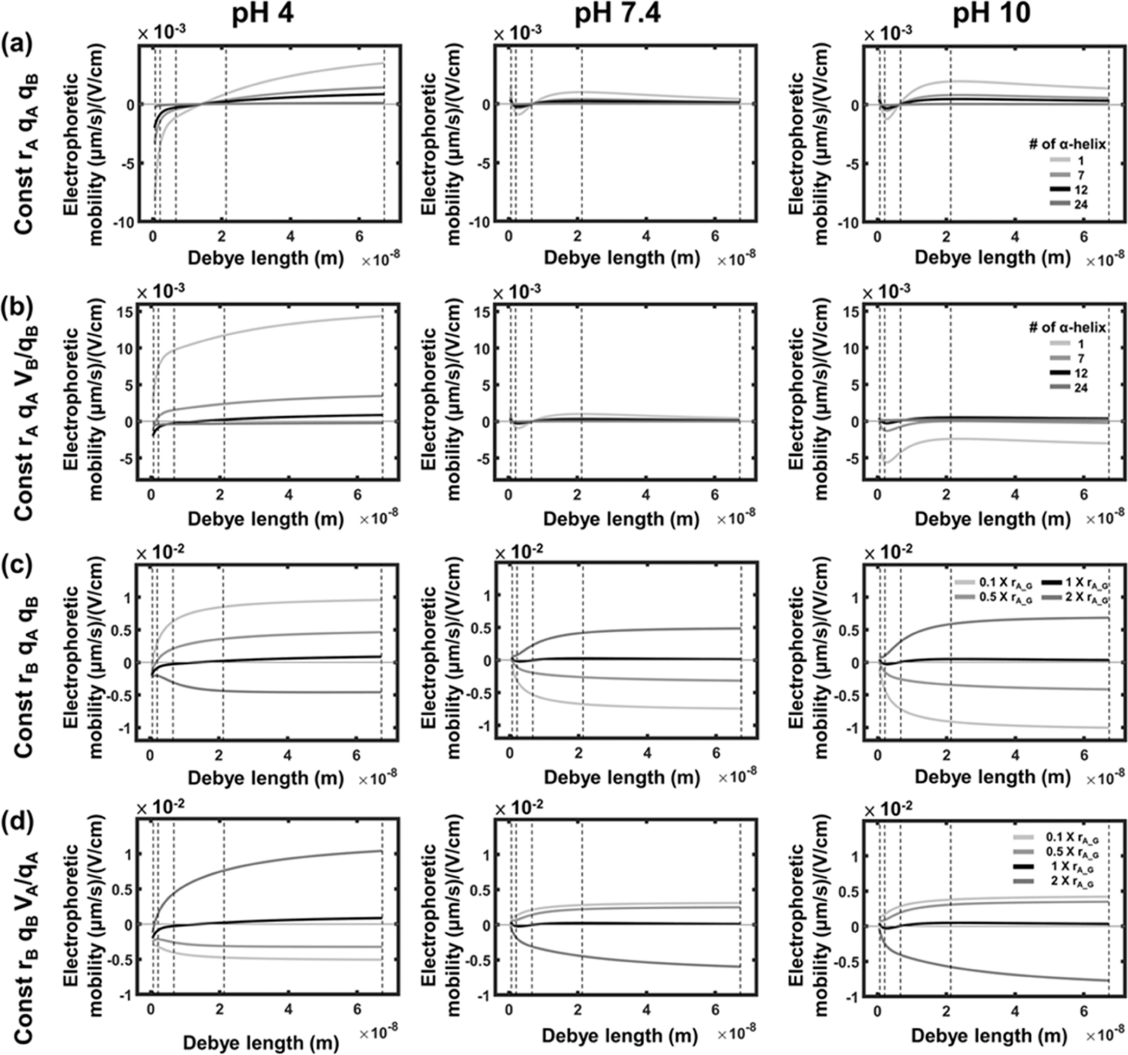
Predicted electrophoretic mobilities of transmembrane proteins at varying (a) *r*_B_ (constant *r*_A_, *q*_A_, *q*_B_), (b) *r*_B_ (constant *r*_A_, *q*_A_, *V*_B_/*q*_B_), (c) *r*_A_ (constant *r*_B_, *q*_A_, *q*_B_), (d) *r*_A_ (constant *r*_B_, *q*_B_, *V*_A_/*q*_A_). The dashed line indicates the Debye length at 1×, 0.1× 0.01×, 0.001×, and 0.0001× PBS (from left to right).

Given the diverse sizes of membrane proteins, we sought to explore the impact of their sizes on the electrophoretic mobility. Figure 7 illustrates the predicted electrophoretic mobility of a transmembrane protein with varying sizes, utilizing surface charges and protein charges obtained from the GLUT1 complex at three pH levels as a reference.

We initially investigated the influence of varying the size of portion B on the electrophoretic mobility, as illustrated in Figure 7a. We considered scenarios where the transmembrane portion consists of 1, 7, 12, and 24 α helices, with corresponding hydrophobic volumes set at 0.08, 0.58, 1, and 2 times the typical volume of the transmembrane portion of the GLUT1 complex. Consequently, *r*_B_ was adjusted to 1.13, 2.98, 3.90, and 5.52 nm, while *q*_A_, *q*_B_, *r*_A_, and σ were held constant. The magnitude of electrophoretic mobility decreases as *r*_B_ increases at all three pH levels, which can be attributed to the increased hydrophobic portion size, causing heightened membrane drag. The mobilities at pH 7.4 are much smaller than those at pH 4 because the magnitude of the net driving force at pH 7.4 is smaller than those at pH 4.

Interestingly, at pH 4, the mobility is negative at small Debye lengths but turns positive at larger Debye lengths. This phenomenon could be explained by the force analysis in Figure 3c, in which the net driving force is a composite of the electroosmotic force, electric force of portion A, and electric force of portion B. The electric force of portion B is assumed to remain unaffected by the Debye length. Both the magnitudes of electroosmotic force and the electric force of portion A increase with Debye length but in opposing directions. At a small Debye length, the dominance of the negative electroosmotic force over the positive electric force of portion A results in negative mobility. On the other hand, at large Debye length, the dominance of the positive electric force of portion A over the negative electroosmotic force results in positive mobility.

Figure 7b explores how the mobility is influenced by *r*_B_ when *q*_B_ varies with the volume of portion B. When *q*_B_ increases, the electric force applied to portion B increases. Because the electric force applied on portion B is assumed to remain unaffected by the Debye length, a constant value is introduced to the net driving force throughout the entire Debye length range in Figure 7b. Consequently, the overall shape and trend of Figure 7b are similar to those of Figure 7a, though there is a noticeable shift that depends on whether the electric force on portion B is increasing or decreasing.

We also explored the impact of varying the size of portion A on the electrophoretic mobility, as depicted in Figure 7c. The predicted electrophoretic mobility of a transmembrane protein was assessed with hydrophilic volumes equal to 0.1, 0.5, 1, and 2 times the typical volume of the GLUT1 complex while maintaining constant values for *q*_A_, *q*_B_, *r*_B_, and σ. The corresponding *r*_*A*_ values were set at 3.4 5.9, 7.4, and 9.3 nm, respectively. Notably, the mobility exhibits distinct behaviors at different pH levels. At pH 4, the mobility transitions from a positive value to a negative one with increasing *r*_A_, while at pH 7.4 and 10, the trend is reversed: shifting from a negative value to a positive value. Analyzing the forces at play shows that when *r*_A_ is small, the electroosmotic force magnitude is small, and the net driving force is predominantly influenced by the electric force of portion A. This dominance results in a positive electrophoretic mobility at pH 4 and a negative mobility at pH 7.4 and 10. As *r*_A_ increases beyond a certain threshold, the electroosmotic force becomes substantial enough to induce a directional switch in the net driving force, consequently altering the mobility direction.

In practical scenarios, the augmentation of *r*_A_ is likely to coincide with an increase in *q*_A_ if we assume a consistent amino acid composition within the expanding volume. Figure 7d offers insights into the predicted electrophoretic mobility of a transmembrane protein as *r*_A_ varies, while the charge-to-volume ratio of portion A (*V*_A_/*q*_A_) is maintained. The trends in Figure 7d are very different from those in Figure 7c, where *q*_A_ is kept constant. The divergence arises from the concurrent increase in *q_A_* with *r*_A_, leading to amplified effects on both hydrodynamic and electric forces of portion A. As *r*_A_ grows, not only does the hydrodynamic force intensify but also the electric force of portion A experiences a rise. If the influence of increasing *r*_A_ on the electric force surpasses its impact on the electroosmotic force, the net driving force is propelled in the direction of the electric force. On the other hand, if the influence of increasing *r*_A_ on the electroosmotic force surpasses its impact on the electric force, the net driving force is propelled in the direction of the electroosmotic force. These result in an increasing trend of positive mobility toward the cathode at pH 4 and a declining trend at pH 7.4 and 10 with increasing *r*_A_.

To comprehensively validate our model, we employed it to predict the electrophoretic mobilities of various lipid probes and proteins across a range of buffer conditions shown in previous studies. We then compared these predictions to their experimentally measured mobilities. Table 2 presents this comparison, including the estimated sizes, charges, and buffer conditions utilized in our analyses. Notably, our predicted electrophoretic mobilities exhibit close alignment with the experimental measurements in previous studies, affirming the robustness and validity of our model.

**Table 2:** Comparison of the Experimentally Measured Electrophoretic Mobilities from Previous Studies with Our Model Predictions

species^a^	buffer type	pH	σ^b^ (mC/m^2^)	λ (nm)	*r*_A_ (nm)	*r*_B_ (nm)	*L* (nm)	*q*_A_	*q*_B_	paper mobility^c^,^d^	predicted mobility^d^	refs.
TR-DHPE (ortho)	1 mM NaH_2_PO_4_ and 5 mM NaCl	4.9	–2.54	3.4	0.5	0.4	2	–1	0	–7.6	–4.3	(12)
TR-DHPE (ortho)	0.5 mM NaH_2_PO_4_ buffer w/o NaCl	4.9	–2.54	10	0.5	0.4	2	–1	0	–6.0	–3.0	
TR-DHPE (para)	1 mM NaH_2_PO_4_ and 5 mM NaCl	4.9	–2.54	3.4	0.3	0.4	2	–1	0	–7.0	–6.3	
TR-DHPE (para)	0.5 mM NaH_2_PO_4_ buffer w/o NaCl	4.9	–2.54	10	0.3	0.4	2	–1	0	–7.0	–5.6	
pR + Alexa488	DI water	N.A.	–1.00	1000	0.5	5.7	5	–2.00	–4.76	–0.3	–0.2	(24)
CymA + ATTO565	DI water	N.A.	–1.00	1000	2	1.8	5	1.11	–0.48	2.2	7.8	(25)
StrA + Alexa488*0.3	0.5 mM sodium citrate/0.5 mM tris buffer + 0 mM NaCl	7.9	–3.50	10	2	0.4	2	–3.07	–2	–1.9	–1.9	
StrA + Alexa488*0.3	0.5 mM sodium citrate/0.5 mM tris buffer + 5 mM NaCl	7.9	–3.50	4	2	0.4	2	–3.07	–2	–5.0	–6.2	
StrA + Alexa488*0.3	0.5 mM sodium citrate/0.5 mM tris buffer + 10 mM NaCl	7.9	–3.50	3	2	0.4	2	–3.07	–2	–5.8	–7.6	
StrA + Alexa488*4	1 mM sodium citrate buffer	4.2	–2.26	11.4	2.4	0.4	2	–3.31	0	6.7	3.4	(16)
StrA + Alexa488*4	1 mM tris buffer	7.5	–3.36	10.1	2.4	0.4	2	–6.14	–2	–6.0	–9.2	
StrA + Alexa488*4	1 mM tris buffer	9.4	–4.10	12.0	2.4	0.4	2	–7.76	–2	–8.8	–9.6	
StrA + Alexa488*0.3	1 mM sodium citrate buffer	4.2	–2.26	11.4	2	0.4	2	0.09	0	9.0	13.2	
StrA + Alexa488*0.3	1 mM tris buffer	7.5	–3.36	10.1	2	0.4	2	–2.74	–2	–1.4	–1.4	
StrA + Alexa488*0.3	1 mM tris buffer	9.4	–4.10	12.0	2	0.4	2	–4.36	–2	–2.5	–2.9	

a TR: Texas Red; pR: proteorhodopsin; StrA: streptavidin.

b Ref (40).

c Electrophoretic mobility from previous studies.

d (×10^–3^ (μm/s)/(V/cm)).

Poyton and Cremer utilized Henry’s function to characterize the electric force acting on two lipid probes, ortho and para Texas Red DHPE.^12^ They observed that their calculated electrophoretic mobilities closely matched the measured mobilities at a Debye length of 3.4 nm. However, a notable discrepancy emerged at a Debye length of 10 nm, where experimentally measured mobilities were smaller compared to the calculated values. This disparity was ascribed to the presence of an electroosmotic force. In our model, which incorporates both electric and electroosmotic forces, we observed that predicted mobilities at a Debye length of 10 nm were indeed smaller than those at a Debye length of 3.4 nm. Our force analysis also clearly shows that the significant presence of the electroosmotic force at larger Debye lengths is indeed the reason for the decreased mobilities.

In addition, Monson et al. conducted experiments, demonstrating that the electrophoretic mobility of a lipid-anchored protein, specifically streptavidin, is notably influenced by both the charge of the labeled streptavidin and pH.^16^ Our model predictions align consistently with the observed trend of how mobilities vary with pH and protein charge at the provided buffer ionic strengths.

For the two transmembrane protein studies, our predictions are consistent with the experimentally measured mobilities. In the case of CymA,^25^ our force analyses elucidate that the large hydrophilic portion induces a substantial electroosmotic force opposing the applied electric forces, resulting in positive mobility, despite the negative charge estimated from the amino acid sequence. In the study using proteorhodopsin,^24^ the observed slow mobility can be attributed to the large portion B and potential trimer formation. While the membrane drag impedes mobility, the net driving force, dominated by electric forces, maintains a negative direction, aligning with the experimental finding. These comprehensive comparisons underscore the reliability and applicability of our model across various experimental scenarios, further validating its utility in elucidating complex electrophoretic phenomena in diverse biological contexts.

## Conclusions

In this study, we showed the tunability of electrophoretic mobility in a 12-transmembrane helix protein, glucose transporter 1 with antibodies (GLUT1 complex), and a lipid probe, Fast-DiO, through manipulation of the buffer pH and ionic strength. The electrophoretic mobility of the GLUT1 complex was observed to be considerably lower than that of Fast-DiO under neutral pH conditions, aligning with the anticipated slow mobility characteristic of transmembrane proteins. Notably, the electrophoretic mobility of the GLUT1 complex exhibited a significant increase at pH 4 and ionic strength exceeding 0.1× PBS. The identified conditions for rapid GLUT1 complex mobility facilitated the efficient separation of GLUT1 and Fast-DiO within a native-like environment in a matter of minutes. More importantly, we constructed a force model that accounts for the distinct electric and drag forces acting on both the transmembrane and aqueous-exposed segments of a transmembrane protein as well as the electroosmotic force to elucidate how the size and charge characteristics of a transmembrane protein can impact its electrophoretic mobility. Importantly, our study highlighted the impact of buffer ionic strength and pH on both the electric force exerted on the hydrophilic portion and the electroosmotic force, consequently affecting the electrophoretic mobility. The model predictions closely matched experimentally measured electrophoretic mobilities of the GLUT1 complex and Fast-DiO across varying pH and ionic strengths. Furthermore, the model fitting parameters provided effective charge information for the hydrophilic and hydrophobic portions of the protein as well as surface charge information, which were justified in this study. Additionally, we applied our model to estimate the mobilities of lipid probes, lipid-anchored proteins, and reconstituted membrane proteins used in previous studies, with predictions aligning well with the reported mobilities. This affirms the applicability of our model to estimating the electrophoretic mobility of diverse transmembrane species.

Considering the diverse sizes of membrane proteins, our model predictions were employed to showcase how variations in hydrophilic and hydrophobic sizes impact mobility. Force analyses revealed that a larger transmembrane portion increases membrane drag, substantially reducing electrophoretic mobility, while a larger hydrophilic portion enhances the electroosmotic force. In our membrane patch system, the electroosmotic force opposes the electric force of the hydrophilic portion. The interplay between electroosmotic and electric forces significantly influences transmembrane protein mobility. If the electric force dominates, then the protein moves in the same direction as its charge. Increasing the hydrophilic portion size increases the opposite electroosmotic force and diminishes mobility. Conversely, if the electroosmotic force dominates, the protein moves in the direction of the electroosmotic flow, and increasing the hydrophilic portion size can amplify mobility. Our findings underscore how the size and charge properties of transmembrane proteins influence the suitable range of operating conditions for efficient movement, presenting potential applications in concentrating and isolating membrane proteins within this platform.

## Supporting Information Available

Method of calculating the center of intensity (Figure S1); calculation of the drift velocity (Figure S2); and calculation of protein charge by amino acid sequence (Figure S3) (PDF)

## Abbreviations

GPMV: giant plasma membrane vesicle
GLUT1: glucose transporter 1
PBS: phosphate buffered saline
PDMS: poly(dimethylsiloxane)
BSA: bovine serum albumin
PFA: paraformaldehyde
DTT: dithiothreitol

## References

[ref1] Conner S. D.; Schmid S. L. Regulated portals of entry into the cell. Nature 2003, 422 (6927)), 37–44. 10.1038/nature01451. 12621426

[ref2] Allen J. A.; Halverson-Tamboli R. A.; Rasenick M. M. Lipid raft microdomains and neurotransmitter signalling. Nat. Rev. Neurosci. 2007, 8 (2), 128–140. 10.1038/nrn2059. 17195035

[ref3] Zhang L.; Zhang Z.; Guo H.; Wang Y. Na+/K+-ATPase-mediated signal transduction and Na+/K+-ATPase regulation. Fundam. Clin. Pharmacol. 2008, 22 (6), 615–621. 10.1111/j.1472-8206.2008.00620.x. 19049666

[ref4] Overington J. P.; Al-Lazikani B.; Hopkins A. L. How many drug targets are there?. Nat. Rev. Drug Discovery 2006, 5 (12), 993–996. 10.1038/nrd2199. 17139284

[ref5] Yang Z.; Wang C.; Zhou Q.; An J.; Hildebrandt E.; Aleksandrov L. A.; Kappes J. C.; DeLucas L. J.; Riordan J. R.; Urbatsch I. L.; et al. Membrane protein stability can be compromised by detergent interactions with the extramembranous soluble domains. Protein Sci. 2014, 23 (6), 769–789. 10.1002/pro.2460. 24652590 PMC4093953

[ref6] Guo Y. Be Cautious with Crystal Structures of Membrane Proteins or Complexes Prepared in Detergents. Crystals 2020, 10 (2), 86 10.3390/cryst10020086. 32494365 PMC7269168

[ref7] Andersen O. S.; Koeppe R. E. Bilayer Thickness and Membrane Protein Function: An Energetic Perspective. Annu. Rev. Biophys. Biomol. Struct. 2007, 36 (1), 107–130. 10.1146/annurev.biophys.36.040306.132643. 17263662

[ref8] Phillips R.; Ursell T.; Wiggins P.; Sens P. Emerging roles for lipids in shaping membrane-protein function. Nature 2009, 459 (7245), 379–385. 10.1038/nature08147. 19458714 PMC3169427

[ref9] Lee A. G. How lipids affect the activities of integral membrane proteins. Biochim. Biophys. Acta, Biomembr. 2004, 1666 (1), 62–87. 10.1016/j.bbamem.2004.05.012. 15519309

[ref10] Groves J. T.; Boxer S. G. Electric field-induced concentration gradients in planar supported bilayers. Biophys. J. 1995, 69 (5), 1972–1975. 10.1016/S0006-3495(95)80067-6. 8580340 PMC1236430

[ref11] Groves J. T.; Wülfing C.; Boxer S. G. Electrical manipulation of glycan-phosphatidyl inositol-tethered proteins in planar supported bilayers. Biophys. J. 1996, 71 (5), 2716–2723. 10.1016/S0006-3495(96)79462-6. 8913608 PMC1233757

[ref12] Poyton M. F.; Cremer P. S. Electrophoretic measurements of lipid charges in supported bilayers. Anal. Chem. 2013, 85 (22), 10803–10811. 10.1021/ac402079e. 24191728 PMC3878813

[ref13] van Weerd J.; Krabbenborg S. O.; Eijkel J.; Karperien M.; Huskens J.; Jonkheijm P. On-chip electrophoresis in supported lipid bilayer membranes achieved using low potentials. J. Am. Chem. Soc. 2014, 136 (1), 100–103. 10.1021/ja411287u. 24345193 PMC3901391

[ref14] Liu C.; Monson C. F.; Yang T.; Pace H.; Cremer P. S. Protein separation by electrophoretic-electroosmotic focusing on supported lipid bilayers. Anal. Chem. 2011, 83 (20), 7876–7880. 10.1021/ac201768k. 21958061 PMC3198849

[ref15] Daniel S.; Diaz A. J.; Martinez K. M.; Bench B. J.; Albertorio F.; Cremer P. S. Separation of membrane-bound compounds by solid-supported bilayer electrophoresis. J. Am. Chem. Soc. 2007, 129 (26), 8072–8073. 10.1021/ja0720816. 17564451 PMC2548333

[ref16] Monson C. F.; Pace H. P.; Liu C.; Cremer P. S. Supported bilayer electrophoresis under controlled buffer conditions. Anal. Chem. 2011, 83 (6), 2090–2096. 10.1021/ac1028819. 21319743 PMC3056904

[ref17] Daniel S.; Chao L. Supported lipid bilayer electrophoresis for separation and analytical studies of cell membrane biomolecules. In Interfaces and Interphases in Analytical Chemistry; ACS Publications, 2011; pp 99–121.

[ref18] Han X.; Cheetham M. R.; Sheikh K.; Olmsted P. D.; Bushby R. J.; Evans S. D. Manipulation and charge determination of proteins in photopatterned solid supported bilayers. Integr. Biol. 2009, 1 (2), 205–211. 10.1039/B815601H. 20023804

[ref19] Stelzle M.; Miehlich R.; Sackmann E. Two-dimensional microelectrophoresis in supported lipid bilayers. Biophys. J. 1992, 63 (5), 1346–1354. 10.1016/S0006-3495(92)81712-5. 19431856 PMC1261439

[ref20] Kam L.; Boxer S. G. Spatially Selective Manipulation of Supported Lipid Bilayers by Laminar Flow: Steps Toward Biomembrane Microfluidics. Langmuir 2003, 19 (5), 1624–1631. 10.1021/la0263413.

[ref21] Pace H. P.; Sherrod S. D.; Monson C. F.; Russell D. H.; Cremer P. S. Coupling supported lipid bilayer electrophoresis with matrix-assisted laser desorption/ionization-mass spectrometry imaging. Anal. Chem. 2013, 85 (12), 6047–6052. 10.1021/ac4008804. 23731179 PMC3717335

[ref22] Grogan M. J.; Kaizuka Y.; Conrad R. M.; Groves J. T.; Bertozzi C. R. Synthesis of lipidated green fluorescent protein and its incorporation in supported lipid bilayers. J. Am. Chem. Soc. 2005, 127 (41), 14383–14387. 10.1021/ja052407f. 16218633

[ref23] Hu S.-K.; Huang L.-T.; Chao L. Membrane species mobility under in-lipid-membrane forced convection. Soft Matter 2016, 12 (33), 6954–6963. 10.1039/C6SM01145D. 27476605

[ref24] Bao P.; Cartron M.; Sheikh K.; Johnson B.; Hunter C.; Evans S. Controlling transmembrane protein concentration and orientation in supported lipid bilayers. Chem. Commun. 2017, 53 (30), 4250–4253. 10.1039/C7CC01023K. 28361139

[ref25] Cheetham M. R.; Bramble J. P.; McMillan D. G. G.; Krzeminski L.; Han X.; Johnson B. R. G.; Bushby R. J.; Olmsted P. D.; Jeuken L. J. C.; Marritt S. J.; Butt J. N.; Evans S. D. Concentrating Membrane Proteins Using Asymmetric Traps and AC Electric Fields. J. Am. Chem. Soc. 2011, 133 (17), 6521–6524. 10.1021/ja2007615. 21476549

[ref26] Cheetham M. R.; Bramble J. P.; McMillan D. G.; Bushby R. J.; Olmsted P. D.; Jeuken L. J.; Evans S. D. Manipulation and sorting of membrane proteins using patterned diffusion-aided ratchets with AC fields in supported lipid bilayers. Soft Matter 2012, 8 (20), 5459–5465. 10.1039/c2sm25473e.

[ref27] Yoshina-Ishii C.; Boxer S. G. Controlling two-dimensional tethered vesicle motion using an electric field: interplay of electrophoresis and electro-osmosis. Langmuir 2006, 22 (5), 2384–2391. 10.1021/la0526277. 16489833 PMC2504470

[ref28] Okamoto Y.; Tsujimoto Y.; Umakoshi H. Electrophoretic separation method for membrane pore-forming proteins in multilayer lipid membranes. Electrophoresis 2016, 37 (5–6), 762–768. 10.1002/elps.201500567. 26773565

[ref29] Gambin Y.; Lopez-Esparza R.; Reffay M.; Sierecki E.; Gov N. S.; Genest M.; Hodges R. S.; Urbach W. Lateral mobility of proteins in liquid membranes revisited. Proc. Natl. Acad. Sci. U.S.A. 2006, 103 (7), 2098–2102. 10.1073/pnas.0511026103. 16461891 PMC1413751

[ref30] Guigas G.; Weiss M. Size-dependent diffusion of membrane inclusions. Biophys. J. 2006, 91 (7), 2393–2398. 10.1529/biophysj.106.087031. 16829562 PMC1562383

[ref31] Quemeneur F.; Sigurdsson J. K.; Renner M.; Atzberger P. J.; Bassereau P.; Lacoste D. Shape matters in protein mobility within membranes. Proc. Natl. Acad. Sci. U.S.A. 2014, 111 (14), 5083–5087. 10.1073/pnas.1321054111. 24706877 PMC3986167

[ref32] Weiß K.; Neef A.; Van Q.; Kramer S.; Gregor I.; Enderlein J. Quantifying the diffusion of membrane proteins and peptides in black lipid membranes with 2-focus fluorescence correlation spectroscopy. Biophys. J. 2013, 105 (2), 455–462. 10.1016/j.bpj.2013.06.004. 23870266 PMC3714877

[ref33] Bag N.; Yap D. H. X.; Wohland T. Temperature dependence of diffusion in model and live cell membranes characterized by imaging fluorescence correlation spectroscopy. Biochim. Biophys. Acta, Biomembr. 2014, 1838 (3), 802–813. 10.1016/j.bbamem.2013.10.009. 24600711

[ref34] Bosmann H. B.; Hagopian A.; Eylar E. H. Cellular membranes: The isolation and characterization of the plasma and smooth membranes of hela cells. Arch. Biochem. Biophys. 1968, 128 (1), 51–69. 10.1016/0003-9861(68)90008-8. 4300292

[ref35] Boullier J. A.; Melnykovych G.; Barisas B. G. A photobleaching recovery study of glucocorticoid effects on lateral mobilities of a lipid analog in S3G HeLa cell membranes. Biochim. Biophys. Acta, Biomembr. 1982, 692 (2), 278–286. 10.1016/0005-2736(82)90532-6. 7171596

[ref36] Cetin B.; Li D. Effect of Joule heating on electrokinetic transport. Electrophoresis 2008, 29 (5), 994–1005. 10.1002/elps.200700601. 18271065

[ref37] Swinney K.; Bornhop D. J. Quantification and evaluation of Joule heating in on-chip capillary electrophoresis. Electrophoresis 2002, 23 (4), 613–620. 10.1002/1522-2683(200202)23:4<613::AID-ELPS613>3.0.CO;2-F. 11870773

[ref38] Henry D. The cataphoresis of suspended particles. Part I.—The equation of cataphoresis. Proc. R. Soc. London, Ser. A 1931, 133 (821), 106–129. 10.1098/rspa.1931.0133.

[ref39] Kang Y.; Li D. Electrokinetic motion of particles and cells in microchannels. Microfluid. Nanofluid. 2009, 6 (4), 431–460. 10.1007/s10404-009-0408-7.

[ref40] Jing D.; Bhushan B. Quantification of Surface Charge Density and Its Effect on Boundary Slip. Langmuir 2013, 29 (23), 6953–6963. 10.1021/la401168w. 23683055

[ref41] Tanaka M.; Hermann J.; Haase I.; Fischer M.; Boxer S. G. Frictional Drag and Electrical Manipulation of Recombinant Proteins in Polymer-Supported Membranes. Langmuir 2007, 23 (10), 5638–5644. 10.1021/la0628219. 17408291

[ref42] McLaughlin S.; Poo M. The role of electro-osmosis in the electric-field-induced movement of charged macromolecules on the surfaces of cells. Biophys. J. 1981, 34 (1), 85–93. 10.1016/S0006-3495(81)84838-2. 6894257 PMC1327455

[ref43] Probstein R. F. Physicochemical Hydrodynamics: An Introduction; John Wiley & Sons, 2005.

[ref44] Liu H.-Y.; Chen W.-L.; Ober C. K.; Daniel S. Biologically Complex Planar Cell Plasma Membranes Supported on Polyelectrolyte Cushions Enhance Transmembrane Protein Mobility and Retain Native Orientation. Langmuir 2018, 34 (3), 1061–1072. 10.1021/acs.langmuir.7b02945. 29020444

[ref45] Richards M. J.; Hsia C.-Y.; Singh R. R.; Haider H.; Kumpf J.; Kawate T.; Daniel S. Membrane Protein Mobility and Orientation Preserved in Supported Bilayers Created Directly from Cell Plasma Membrane Blebs. Langmuir 2016, 32 (12), 2963–2974. 10.1021/acs.langmuir.5b03415. 26812542

[ref46] Lyu S.-W.; Wang J.-F.; Chao L. Constructing supported cell membranes with controllable orientation. Sci. Rep. 2019, 9 (1), 2747 10.1038/s41598-019-39075-8. 30808885 PMC6391389

[ref47] Kuo C.-W.; Kuo T.-H.; Lee H.-A.; Lin Y.-T.; Kuo C.-J.; Hsiao K.-H.; Yang M.-H.; Tanady K.; Chang S.-Y.; Lin T.-R.; Chao L. Label-free detection of transport kinetics and inhibitor binding of membrane transport proteins with a two-mode plasmonic sensor. Biosens. Bioelectron.: X 2022, 11, 100183 10.1016/j.biosx.2022.100183.

[ref48] Setareh Biotech/product 6297. https://www.setarehbiotech.com/Setareh/pub_web/showProductPubWeb.action;jsessionid=94D8764D89E539FD818440034FCAC856?productId=1594 (accessed November 16, 2023).

[ref49] Jönsson P.; Beech J. P.; Tegenfeldt J. O.; Höök F. Shear-Driven Motion of Supported Lipid Bilayers in Microfluidic Channels. J. Am. Chem. Soc. 2009, 131 (14), 5294–5297. 10.1021/ja809987b. 19309139

[ref50] Jönsson P.; Höök F. Effects of Surface Pressure and Internal Friction on the Dynamics of Shear-Driven Supported Lipid Bilayers. Langmuir 2011, 27 (4), 1430–1439. 10.1021/la103959w. 21142022

[ref51] Ratajczak A. M.; Sasidharan S.; Gonzalez X. I. R.; Miller E. J.; Socrier L.; Anthony A. A.; Honerkamp-Smith A. R. Measuring flow-mediated protein drift across stationary supported lipid bilayers. Biophys. J. 2023, 122 (9), 1720–1731. 10.1016/j.bpj.2023.03.042. 37020419 PMC10183372

[ref52] Hughes M. P.; Hoettges K. F.; Hughes M. P. Microengineering in biotechnology; Springer, 2010.

[ref53] Deen W. M. Analysis of Transport Phenomena. Oxford university press: New York, 1998 2.

[ref54] Wiersema P. H.; Loeb A. L.; Overbeek J. T. G. Calculation of the electrophoretic mobility of a spherical colloid particle. J. Colloid Interface Sci. 1966, 22 (1), 78–99. 10.1016/0021-9797(66)90069-5.

[ref55] Young H. D.; Freedman R. A.; Ford A. L. Sears and Zemansky’s University Physics: With Modern Physics; Addison-Wesley, 2012.

[ref56] Ohshima H. A simple expression for Henry’s function for the retardation effect in electrophoresis of spherical colloidal particles. J. Colloid Interface Sci. 1994, 168 (1), 269–271. 10.1006/jcis.1994.1419.

[ref57] Bird R. B.; Stewart W. E.; Lightfoot E. N. Transport phenomena/R. Byron Bird, Warren E. Stewart, Edwin N. Lightfoot. 2nd ed.; J. Wiley: New York, 2002.

[ref58] Xia X. Protein isoelectric point. In Bioinformatics and the Cell: Modern Computational Approaches in Genomics, Proteomics and Transcriptomics; Springer, 2007; pp 207–219.

[ref59] Henriksson G.; Englund A. K.; Johansson G.; Lundahl P. Calculation of the isoelectric points of native proteins with spreading of pKa values. Electrophoresis 1995, 16 (1), 1377–1380. 10.1002/elps.11501601227. 8529600

[ref60] Bateman A.; Martin M. J.; Orchard S.; et al. UniProt: the universal protein knowledgebase in 2021. Nucleic Acids Res. 2020, 49 (D1), D480–D489. 10.1093/nar/gkaa1100. PMC7778908 33237286

[ref61] Yang D.; Kroe-Barrett R.; Singh S.; Laue T. IgG Charge: Practical and Biological Implications. Antibodies 2019, 8, 24 10.3390/antib8010024. 31544830 PMC6640702

[ref62] Zumdahl S. S.; DeCoste D. J. Chemical Principles; Cengage Learning, 2012.

