# Supplementary material for: Rapid Enrichment of a Native Multipass Transmembrane Protein via Cell Membrane Electrophoresis through Buffer pH and Ionic Strength Adjustment
Source: J Am Chem Soc. 2024 Apr 17;146(17):11634–47. doi: 10.1021/jacs.3c13579 (PMC11066866; doi:10.1021/jacs.3c13579)
Supplement: Supplementary file 1 — ja3c13579_si_001.pdf [file ja3c13579_si_001.pdf]

Supplementary Information

Rapid Enrichment of a Native Multipass Transmembrane Protein via Cell Membrane Electrophoresis through Buffer pH and Ionic Strength Adjustment

Tzu-Tzu Liu, Sin-Han Huang, Ling Chao*

Department of Chemical Engineering, National Taiwan University, Taipei, Taiwan
*Correspondence should be addressed to L.C. (lingchao@ntu.edu.tw)

Table of contents:

1. Method of Calculating the Center of Intensity and the Displacement of the Center of Intensity. **(Figure S1)**	S2
2. Calculation of Drift Velocity. **(Figure S2)**	S3
3. Calculation of Protein Charge by Amino Acid Sequence. **(Figure S3) (Table S1, S2)**	S4
4. Properties of Membrane Species Used in the Model **(Table S3, S4)**	S6
5. Comparison of Predicted Mobility using our Model with Previous Studies. **(Table S5)**	S8
References	S10

1. Method of Calculating the Center of Intensity and the Displacement of the Center of Intensity

We employed the methodology from our prior investigation[1] to compute the center of intensity within a given patch. Our approach assumed that the intensity of each pixel correlates with the concentration of the target species, thereby defining the center of intensity as the center of mass for the species within the patch. Aligning with the x-direction of our chosen image as the electric field direction and the perpendicular as the y-direction, the x and y positions of the center of intensity were determined using the following equations, as illustrated in Figure S1(a):

$$\frac{\sum_{y=1}^{y=n}((\sum_{x=1}^{x=m} \mathrm{I(x,y)})\cdot \mathrm{y})}{\sum_{y=1}^{y=n}(\sum_{x=1}^{x=m} \mathrm{I(x,y)})} = center\ of\ intensity\ in\ y\ direction \quad \text{(S1)}$$

$$\frac{\sum_{x=1}^{x=m}\left((\sum_{y=1}^{y=n} \mathrm{I(x,y)})\cdot \mathrm{x}\right)}{\sum_{x=1}^{x=m}(\sum_{y=1}^{y=n} \mathrm{I(x,y)})} = center\ of\ intensity\ in\ x\ direction \quad \text{(S2)}$$

where I(x,y) is the intensity at a certain position.

The center of intensity's position was then established as the origin for new axes to facilitate the selection of an appropriate region for subsequent data analyses. To ensure inclusivity of intensity at the boundary, post center of intensity determination, we extended 1.2 times the distance to the boundary from the center in both left and right directions along the x-axis. The height was extended above and below the center by 0.05 times the height of the membrane patch. The chosen analyzed region is depicted as the yellow rectangular area in Figure S1(b). Subsequently, we conducted an averaging of intensity in the y-direction to convert the intensity within the analyzed region into one dimension.

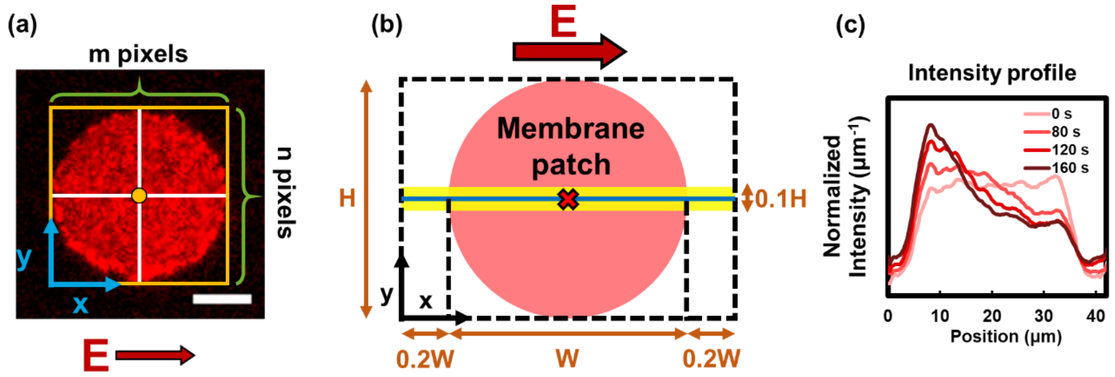

Figure S1. (a) Illustration depicting the definition of the center of intensity and the chosen region within the membrane patch. (b) Schematic diagram illustrating the selected region (highlighted in yellow) for subsequent analyses. (c) Intensity profile derived from the selected region.

Due to the photobleaching of fluorescent molecules, the image intensity diminished over time. To mitigate the impact of photobleaching, we normalized the intensity profiles based on the conservation of species mass within the chosen region. Our prior research demonstrated negligible diffusion mass flux across the upper and lower boundaries of the selected rectangular box, and at the patch boundaries, no mass flux was observed[1]. Consequently, we assumed mass conservation within the selected box, normalizing the sum of intensities of all pixels to one in this study. The normalized intensities in Figure 1(b), (c), and Figure S1(c) are calculated using Equation (S3):

$$\mathrm{I}_{N\_b}(\mathrm{x}) = \frac{\mathrm{I(x)}}{\sum_{x=1}^{x=m} \mathrm{I(x)}} \quad \text{(S3)}$$

To determine the displacement of the center of intensity, we calculated the center of intensity in the x-direction within the selected box using Equation (S4). The displacement of the center of intensity at a specific time was determined by subtracting the location of the center of intensity at t=0 from the location at the particular time of interest.

$$\frac{\sum \mathrm{I}_{N_b}(X)*x}{\sum \mathrm{I}_{N\_b}(X)} = \text{the center of intensity in the selected box in the x direction} \quad \text{(S4)}$$

2. Calculation of the Drift Velocity

We determined the drift velocity by examining the slope of the displacement-time curve for the intensity's center. Figure 1(b) illustrates the typical displacement-time curves for the GLUT1-complex in 0.1X PBS at pH levels 4, 7.4, and 10. At pH 4, the displacement curve undergoes a marked alteration, while it remains relatively unchanged at pH 7.4 and pH 10. Consequently, we utilized the behavior at pH 4 to delineate the time interval of interest for conducting steady-state force analysis. The pH 4 graph displays a brief initial phase, followed by a significant shift within the first 100-200 seconds, and then transitions into a slower rate of change. This behavior indicates that the migration times of small and large membrane entities differ. Initially, the swift accumulation of small components (such as charged lipids) possibly creates an additional electric field, influencing the movement of the slower large membrane proteins. The latter period, marked by a gradual shift or a plateau, is primarily due to the restrictions set by the patch boundary. The significant linear displacement noted during the mid-phase reflects a pseudo-steady state condition, wherein the electric force, prompted by the distribution of other membrane entities, is nearly constant. Consequently, the overall force exerted on the GLUT1-complex essentially balances out to zero.

In our analysis of drift velocity, we concentrated on the notable linear displacement occurring in the mid-phase. To accurately assess this phase, we performed linear regression in intervals, using sets of ten data points each, to identify the interval that produced the highest R-squared (R^2) value, indicative of the strongest linear relationship. This method facilitated the identification of the most appropriate slope for the displacement-time curve during the pseudo-steady state phase. We observed that, under certain conditions, an initial startup phase presented a slope different from that of the subsequent longer pseudo-steady state. Typically, these startup phases lasted less than 30 seconds. Therefore, by applying linear fitting to segments of approximately 150 seconds (10 data points), we effectively minimized the influence of the differing slopes observed during these brief initial phases, ensuring a focus on the slope representative of the pseudo-steady state. We also applied the same way to analyze the curves at pH levels 7.4, and 10.

Moreover, our system comprises a membrane patch with defined boundaries. To account for this, we introduced the 'effective mobile fraction' concept, distinguishing the portion of the system actively contributing to migration. This was determined by comparing the normalized intensity profiles at the initial (t=0) and final stationary states (t=ts), as illustrated in Figure S2. Upon reaching a stationary state, a discernible fraction of the species transitions from the grey area to the green area. We define this migratory fraction (S_{mobile}) over the total calculated for the mass center (S_{mobile} + $S_{immobile}$) as the effective mobile fraction. Drift velocity is thus calculated by dividing the mass center migration speed by the effective mobile fraction, as detailed in Eq. S5

$$drift\ velocity = \frac{migration\ speed\ of\ the\ mass\ center}{effective\ mobile\ fraction} \quad \text{(S5)}$$

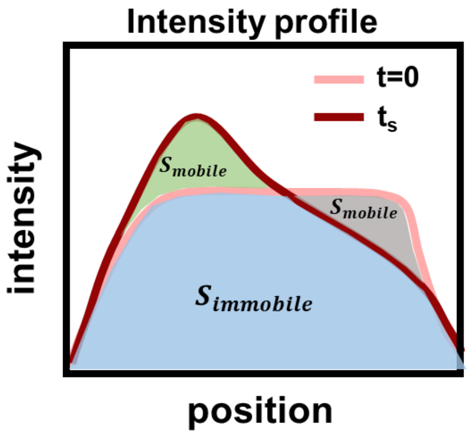

Figure S2. Normalized intensity profile comparison between initial (t=0) and final stationary states (t=ts). S_{mobile} represents the actively migrating component within the membrane patch system. Initially, S_{mobile} localized within the grey area at t=0 and relocated to the green area at t=ts. The overall mass used to calculate the mass center includes the summation of S_{mobile} and $S_{immobile}$.

3. Calculation of Protein Charge by the Amino Acid Sequence

The net positive and negative charge of a protein can be calculated using the Henderson–Hasselbalch equation, as demonstrated in Equations (S6) and (S7)[2]. The net charge is the sum of the positive and negative charges. Figure S3 illustrates the calculated net charge of different regions of GLUT1 at various pH values.

$$\text{positive charge} = \sum\left(\frac{1}{1+10^{(pKa_i - pH)}} \times n_i\right) \quad (n_i = number\ of\ amino\ acid\ H, K, R) \tag{S6}$$

$$\text{negative charge} = \sum\left(\frac{1}{1+10^{(pH - pKa_i)}} \times n_i\right) \quad (n_i = number\ of\ amino\ acid\ C, D, E, Y) \tag{S7}$$

Here, n_i represents the number of amino acids (H, K, R for positive charge; C, D, E, Y for negative charge), the amino acid sequence of GLUT1 is provided in Table S1, and the pKa values of various amino acids are detailed in Table S2.

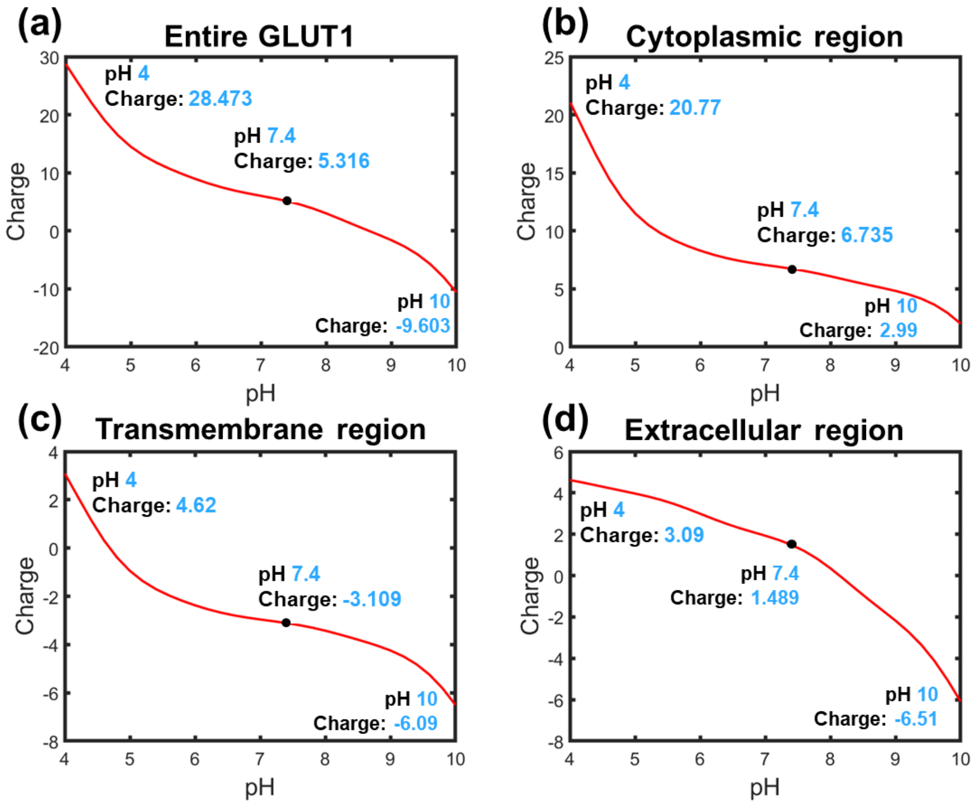

Figure S3. The calculated net charge of GLUT1 at various pH values. The calculation is based on the sequence of (a) the entire GLUT1 molecule, (b) the cytoplasmic region, (c) the transmembrane region, and (d) the extracellular region.

Table S1. The amino sequence of GLUT1[3].

Region	Position(s)	Sequence
cytoplasmic	1~11	MEPSSKKLTGR
transmembrane	12~33	LMLAVGGAVLGSLQFGYNTGVI
extracellular	34~66	NAPQKVIEEFYNQTWVHRYGESILPTTLTTLWS
transmembrane	67~87	LSVAIFSVGGMIGSFSVGLFV
cytoplasmic	88~90	NRF
transmembrane	91~112	GRRNSMLMMNLLAFVSAVLMGF
extracellular	113~120	SKLGKSFE
transmembrane	121~144	MLILGRFIIGVYCGLTTGFVPMYV
cytoplasmic	145~155	GEVSPTALRGA
transmembrane	156~176	LGTLHQLGIVVGILIAQVFGL
extracellular	177~185	DSIMGNKDL
transmembrane	186~206	WPLLLSIIFIPALLQCIVLPF
cytoplasmic	207~271	CPESPRFLLINRNEENRAKSVLKKLRGTADVTHDLQEMKEES RQMMREKKVTILELFRSPAYRQP
transmembrane	272~293	ILIAVVLQLSQQLSGINAVFYY

extracellular	294~306	STSIFEKAGVQQP
transmembrane	307~328	VYATIGSGIVNTAFTVVSLFVV
cytoplasmic	329~334	ERAGRR
transmembrane	335~355	TLHLIGLAGMAGCAILMTIAL
extracellular	356~365	ALLEQLPWMS
transmembrane	366~388	YLSIVAIFGFVAFFEVGPGPIPW
cytoplasmic	389~401	FIVAELFSQGPRP
transmembrane	402~422	AAIAVAGFSNWTSNFIVGMCF
extracellular	423~429	QYVEQLC
transmembrane	430~450	GPYVFIIFTVLLVLFFIFTYF
cytoplasmic	451~492	KVPETKGRTFDEIASGFRQGGASQSDKTPEELFHPLGADSQV

Table S2. The pKa values of the amino acid residues and terminal groups.

Amino acid residues or terminal groups	pKa values	Reference
Terminal carboxyl	3.60	4
Glutamate	4.25	5
Aspartate	3.65	5
Cysteine	8.18	5
Tyrosine	10.07	5
Terminal amino	8.0	4
Histidine	6.00	5
Lysine	10.53	5
Arginine	12.48	5

4. Properties of Membrane Species Used in the Model

Table S3 presents the properties utilized in the model for GLUT1-complex and Fast-DiO. In this context, y is defined as the distance from the substrate surface to the center of portion A, and in this study, we set it as 5 nm+r_A, with 5 nm accounting for the cell membrane thickness[6-7]. Assuming one primary and one secondary antibody bind to one GLUT1, we estimated r_{A_GLUT1} as 7.4 nm, derived from the rotation

radius of the antibody dimer[8]. The estimated q_A includes the charge of the cytoplasmic part of GLUT1, estimated from the sequence, plus the charges of two IgG antibodies.

Table S3. The properties used in the model fitting.

Symbol	Property	Unit	Value	Annotation	Ref.
ε_0	Vacuum permittivity	$CV^{-1}m^{-1}$	$8.854*10^{-12}$	-	9
ε_r	Dielectric constant of water at 25°C	-	78.408	Approximation of diluted PBS	9
**k**	Boltzmann's constant	JK^{-1}	$1.38*10^{-23}$	-	9
**T**	Temperature	K	298.15	-	This study
**e**	Elementary charge	C	$1.6*10^{-19}$	-	9
N_A	Avogadro constant	mol^{-1}	$6.02*10^{23}$	-	9
**E**	Electric field	V/m	3846	-	This study
μ	Viscosity of water at 20°C	Pa s	0.001	Approximation of diluted PBS	9
**η**	Viscosity of cell membrane	Pa s	0.228	-	10
**y**	Distance from substrate to center of portion A	nm	$5+r_A$	Estimated from cell membrane thickness	6-7
r_{A_GLUT1}	Radius of GLUT1 portion A	nm	7.4	Estimation based on dimensions of antibody complex	8, 11
r_{B_GLUT1}	Radius of GLUT1 portion B	nm	3.9	Estimation based on Stokes radius of GLUT1-detergent monomer	12
$r_{A\_Fast\text{-}DiO}$	Radius of Fast-DiO portion A	nm	0.5		
$r_{B\_Fast\text{-}DiO}$	Radius of Fast-DiO portion B	nm	0.4		13-14
$L_{\_GLUT1}$	Height of GLUT1 portion B	nm	5		
$L_{\_Fast\text{-}DiO}$	Height of Fast-DiO portion B	nm	2		

Table S4. The electric properties of GLUT1 complex estimated from the amino acid sequence and literature report.

	pH 4	pH 7.4	pH 10
Cytoplasmic region net charge	41.54	13.51	5.98
Transmembrane region net charge	9.24	-6.22	-12.18
Extracellular region net charge	6.18	2.98	-13.02

IgG net charge[15]	7.05 (pH 5)	-7.11	-
IgG*2+ Cytoplasmic net charge	55.64	-0.71	< -20.2

5. Comparison of Predicted Mobility Using Our Model with Previous Studies

Table S6 shows the predicted electrophoretic mobility of the membrane species shown in previous studies. Since the lipid membrane used in these studies were artificial membranes without cholesterol, the η used to calculate the mobility is 0.12 Pa*s[16]. The Debye length was estimated from the electrophoretic buffer type used in the experiments, if not explicitly mentioned. Surface charge densities were derived from the glass surface charge density at various pH values and different ionic strengths[17].

The net charges of transmembrane proteins, proteorhodopsin (pR) and CymA, were calculated using our model's fitting result at pH 7.4, by assuming V_A/q_A=const and V_B/q_B=const. The r_A of CymA was estimated from the PDB model. CymA has a single alpha-helix in the transmembrane region, and the r_B is estimated as the hydrodynamic radius of a single alpha-helix. Proteorhodopsin, predominantly composed of a transmembrane portion with small cytoplasmic and extracellular regions, has its r_A estimated using the size of the fluorescence dye, Alexa-488. The mobile pR protein was reported predominantly as trimer[18]. With the monomer has 7 alpha-helices in the transmembrane region and considering its reported trimeric form, the r_B was estimated based on 21 alpha-helices.

The q_A of streptavidin was estimated from the charges of streptavidin at different pH value[19], along with the charge of Alexa-488, assumed to be -2 per molecule from its molecular structure. The q_B could originate from biotin on the lipid, with the charge of biotin set as -1 at pH 7.5 and pH 9.4, and as no charge at pH 4.2. Given that one streptavidin molecule often binds to two biotins on the supported lipid membrane[20-21], the q_B was set as -2 at pH 7.5 and pH 9.4, and 0 at pH 4.2. The drag force inside the membrane was also set as two times the drag of a single biotin-lipid molecule.

Table S5. The parameters used to predict the electrophoretic mobility of the membrane species shown in previous studies.

Species [a]	Buffer type	pH	σ [b] (mC/m²)	λ (nm)	r_A (nm)	r_B (nm)	L (nm)	q_A	q_B	Paper mobility [c,d]	Predicted mobility [d]	E [e]	F_{EO} [f]	F_{EA} [f]	F_{EB} [f]	$F_{Driving}$ [f]	Ref.
TR-DHPE (ortho)	1 mM NaH_2PO_4 and 5 mM NaCl	4.9	-2.54	3.4	0.5	0.4	2	-1	0	-7.6	-4.3	86.7	0.57	-1.21	0	-0.65	24
TR-DHPE (ortho)	0.5 mM NaH_2PO_4 buffer w/o NaCl	4.9	-2.54	10	0.5	0.4	2	-1	0	-6.0	-3.0	86.7	0.88	-1.32	0	-0.44	
TR-DHPE (para)	1 mM NaH_2PO_4 and 5 mM NaCl	4.9	-2.54	3.4	0.3	0.4	2	-1	0	-7.0	-6.3	86.7	0.33	-1.28	0	-0.94	
TR-DHPE (para)	0.5 mM NaH_2PO_4 buffer w/o NaCl	4.9	-2.54	10	0.3	0.4	2	-1	0	-7.0	-5.6	86.7	0.51	-1.35	0	-0.83	
pR +Alexa488	DI water	N.A.	-1.00	1000	0.5	5	5	-2.00	-4.76	-0.3	-0.2	66	0.26	-1.59	-3.81	-5.15	18
CymA+ ATTO565	DI water	N.A.	-1.00	1000	2	1.8	5	1.11	-0.48	2.2	7.8	50	1.30	0.88	0.36	2.53	23
StrA+ Alexa488*0.3	0.5 mM sodium citrate/0.5 mM Tris buffer + 0 mM NaCl	7.9	-3.5	10	2	0.4	2	-3.07	-2	-1.9	-1.9	170	9.30	-5.74	-4.48	-0.93	22
StrA + Alexa488*0.3	0.5 mM sodium citrate/0.5 mM Tris buffer + 5 mM NaCl	7.9	-3.5	4	2	0.4	2	-3.07	-2	-5.0	-6.2	170	6.11	-4.65	-4.48	-3.02	
StrA + Alexa488*0.3	0.5 mM sodium citrate/0.5 mM Tris buffer + 10 mM NaCl	7.9	-3.5	3	2	0.4	2	-3.07	-2	-5.8	-7.6	170	5.00	-4.21	-4.48	-3.69	
StrA + Alexa488*4	1mM sodium citrate buffer	4.2	-2.26	11.4	2.4	0.4	2	-3.31	0	6.7	3.4	170	9.46	-7.46	0	2.00	
StrA + Alexa488*4	1mM Tris buffer	7.5	-3.36	10.1	2.4	0.4	2	-6.14	-2	-6.0	-9.2	170	13.56	-13.55	-5.44	-5.43	
StrA + Alexa488*4	1mM Tris buffer	9.4	-4.1	12.0	2.4	0.4	2	-7.76	-2	-8.8	-9.6	170	17.41	-17.63	-5.44	-5.66	
StrA + Alexa488*0.3	1mM sodium citrate buffer	4.2	-2.26	11.4	2	0.4	2	0.09	0	9.0	13.2	170	7.57	0.22	0	7.79	
StrA + Alexa488*0.3	1mM Tris buffer	7.5	-3.36	10.1	2	0.4	2	-2.74	-2	-1.4	-1.4	170	10.88	-6.24	-5.44	-0.80	
StrA + Alexa488*0.3	1mM Tris buffer	9.4	-4.1	12.0	2	0.4	2	-4.36	-2	-2.5	-2.9	170	13.93	-10.18	-5.44	-1.69	

[a] TR : Texas Red.; pR : proteorhodopsin; StrA : streptavidin. [b] Ref. [17]. [c] Electrophoretic mobility from previous studies. [d] ($x10^{-3}$ (μm/s)/(V/cm)). [e](V/cm). [f]($x10^{-15}$ (N))

References

1. Huang, S.-H.; Huang, B.-C.; Chao, L., Development of Cell Membrane Electrophoresis to Measure the Diffusivity of a Native Transmembrane Protein. *Analytical Chemistry* **2022,** *94* (10), 4531-4537.
2. Lunkad, R.; Murmiliuk, A.; Tošner, Z.; Štěpánek, M.; Košovan, P., Role of pKA in Charge Regulation and Conformation of Various Peptide Sequences. *Polymers* **2021,** *13* (2), 214.
3. Consortium, T. U., UniProt: the universal protein knowledgebase in 2021. *Nucleic Acids Research* **2020,** *49* (D1), D480-D489.
4. Matthew, J. B., Electrostatic effects in proteins. *Annual review of biophysics and biophysical chemistry* **1985,** *14* (1), 387-417.
5. Lehninger, A. L.; Nelson, D. L.; Cox, M. M.; Cox, M. M., *Lehninger principles of biochemistry*. Macmillan: 2005.
6. Regan, D.; Williams, J.; Borri, P.; Langbein, W., Lipid Bilayer Thickness Measured by Quantitative DIC Reveals Phase Transitions and Effects of Substrate Hydrophilicity. *Langmuir* **2019,** *35* (43), 13805-13814.
7. Arinaminpathy, Y.; Khurana, E.; Engelman, D. M.; Gerstein, M. B., Computational analysis of membrane proteins: the largest class of drug targets. *Drug Discov Today* **2009,** *14* (23-24), 1130-5.
8. Yearley, Eric J.; Godfrin, Paul D.; Perevozchikova, T.; Zhang, H.; Falus, P.; Porcar, L.; Nagao, M.; Curtis, Joseph E.; Gawande, P.; Taing, R.; Zarraga, Isidro E.; Wagner, Norman J.; Liu, Y., Observation of Small Cluster Formation in Concentrated Monoclonal Antibody Solutions and Its Implications to Solution Viscosity. *Biophysical Journal* **2014,** *106* (8), 1763-1770.
9. Haynes, W. M., *CRC handbook of chemistry and physics, 95th Edition*. 95th ed.; CRC Press: Hoboken, 2014.
10. Kubankova, M.; Lopez-Duarte, I.; Kiryushko, D.; Kuimova, M. K., Molecular rotors report on changes in live cell plasma membrane microviscosity upon interaction with beta-amyloid aggregates. *Soft Matter* **2018,** *14* (46), 9466-9474.
11. Tan, Y. H.; Liu, M.; Nolting, B.; Go, J. G.; Gervay-Hague, J.; Liu, G.-y., A Nanoengineering Approach for Investigation and Regulation of Protein Immobilization. *ACS Nano* **2008,** *2* (11), 2374-2384.
12. Hebert, D. N.; Carruthers, A., Glucose transporter oligomeric structure determines transporter function. Reversible redox-dependent interconversions of tetrameric and dimeric GLUT1. *Journal of Biological Chemistry* **1992,** *267* (33), 23829-23838.
13. Hills Jr, R. D.; McGlinchey, N., Model parameters for simulation of physiological lipids. *Journal of computational chemistry* **2016,** *37* (12), 1112-1118.
14. PubChem Compound Summary for CID 2762625, 3,3'-Dioctadecyloxacarbocyanine perchlorate. https://pubchem.ncbi.nlm.nih.gov/compound/3_3_-Dioctadecyloxacarbocyanine-perchlorate (accessed 2023-11-15).
15. Yang, D.; Kroe-Barrett, R.; Singh, S.; Laue, T. IgG Charge: Practical and Biological Implications *Antibodies* [Online], 2019, p. 1-18.
16. Cogan, U.; Shinitzky, M.; Weber, G.; Nishida, T., Microviscosity and order in the hydrocarbon region of phospholipid and phospholipid-cholesterol dispersions determined with fluorescent probes. *Biochemistry* **1973,** *12* (3), 521-528.

17. Jing, D.; Bhushan, B., Quantification of Surface Charge Density and Its Effect on Boundary Slip. *Langmuir* **2013,** *29* (23), 6953-6963.
18. Bao, P.; Cartron, M.; Sheikh, K.; Johnson, B.; Hunter, C.; Evans, S., Controlling transmembrane protein concentration and orientation in supported lipid bilayers. *Chemical Communications* **2017,** *53* (30), 4250-4253.
19. Sivasankar, S.; Subramaniam, S.; Leckband, D., Direct molecular level measurements of the electrostatic properties of a protein surface. *Proceedings of the National Academy of Sciences* **1998,** *95* (22), 12961-12966.
20. Lou, C.; Wang, Z.; Wang, S.-W., Two-Dimensional Protein Crystals on a Solid Substrate: Effect of Surface Ligand Concentration. *Langmuir* **2007,** *23* (19), 9752-9759.
21. Schmidt, A.; Spinke, J.; Bayerl, T.; Sackmann, E.; Knoll, W., Streptavidin binding to biotinylated lipid layers on solid supports. A neutron reflection and surface plasmon optical study. *Biophysical journal* **1992,** *63* (5), 1385-1392.
22. Monson, C. F.; Pace, H. P.; Liu, C.; Cremer, P. S., Supported bilayer electrophoresis under controlled buffer conditions. *Anal Chem* **2011,** *83* (6), 2090-2096.
23. Cheetham, M. R.; Bramble, J. P.; McMillan, D. G. G.; Krzeminski, L.; Han, X.; Johnson, B. R. G.; Bushby, R. J.; Olmsted, P. D.; Jeuken, L. J. C.; Marritt, S. J.; Butt, J. N.; Evans, S. D., Concentrating Membrane Proteins Using Asymmetric Traps and AC Electric Fields. *Journal of the American Chemical Society* **2011,** *133* (17), 6521-6524.
24. Poyton, M. F.; Cremer, P. S., Electrophoretic measurements of lipid charges in supported bilayers. *Anal Chem* **2013,** *85* (22), 10803-10811.
